# An integrative structural study of the human full-length RAD52 at 2.2 Å resolution

**DOI:** 10.1038/s42003-024-06644-1

**Published:** 2024-08-08

**Authors:** Beatrice Balboni, Roberto Marotta, Francesco Rinaldi, Giulia Milordini, Giulia Varignani, Stefania Girotto, Andrea Cavalli

**Affiliations:** 1 Computational and Chemical Biology, Istituto Italiano di Tecnologia, Genoa, Italy; 2 Department of Pharmacy and Biotechnology, University of Bologna, Bologna, Italy; 3 Electron Microscopy Facility (EMF), Istituto Italiano di Tecnologia, Genoa, Italy; 4 Structural Biophysics Facility, Istituto Italiano di Tecnologia, Genoa, Italy; 5 CECAM, Swiss Federal Institute of Technology Lausanne (EPFL), Lausanne, Switzerland

**Keywords:** Drug discovery, Cryoelectron microscopy, SAXS, Drug development, Intrinsically disordered proteins

## Abstract

Human RAD52 (RAD52) is a DNA-binding protein involved in many DNA repair mechanisms and genomic stability maintenance. In the last few years, this protein was discovered to be a promising novel pharmacological target for anticancer strategies. Although the interest in RAD52 has exponentially grown in the previous decade, most information about its structure and mechanism still needs to be elucidated. Here, we report the 2.2 Å resolution cryo-EM reconstruction of the full-length RAD52 (FL-RAD52) protein. This allows us to describe the hydration shell of the N-terminal region of FL-RAD52, which is structured in an undecamer ring. Water molecules coordinate with protein residues to promote stabilization inside and among the protomers and within the inner DNA binding cleft to drive protein-DNA recognition. Additionally, through a multidisciplinary approach involving SEC-SAXS and computational methods, we comprehensively describe the highly flexible and dynamic organization of the C-terminal portion of FL-RAD52. This work discloses unprecedented structural details on the FL-RAD52, which will be critical for characterizing its mechanism of action and inhibitor development, particularly in the context of novel approaches to synthetic lethality and anticancer drug discovery.

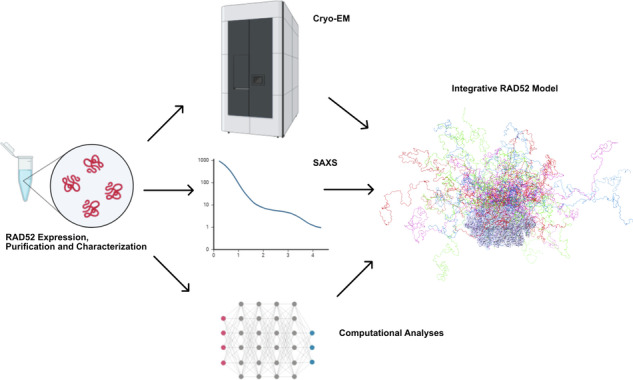

## Abstract (web summary)

A cryo-EM reconstruction with 2.2 Å resolution of the full-length RAD52 protein highlights the hydration shell of the DNA-binding region and the highly flexible C-terminal portion, obtained by combining SEC-SAXS and computational methods.

## Introduction

Human RAD52 (RAD52) is a DNA-binding protein that is assumed to be involved in several DNA repair mechanisms such as Single Strand Annealing (SSA)^1–4^, Homologous Recombination (HR)^5–8^, stalled replication fork protection and processing^9,10^ RNA-dependent DNA repair^11–13^ which are activated throughout the cell cycle to control genome stability maintenance^11,14–16^. Nevertheless, RAD52 has long been considered non-essential since it primarily plays an ancillary role in promoting RAD51-mediated DNA repair or serves as a backup mediator at specific cell cycle time points^17,18^. Only ten years ago, when RAD52 depletion was observed to induce synthetic lethality in BRCA2, BRCA1, and PALB2 mutated cancer cells^19–21^, its potential for anticancer therapy development began to be understood. Since then, many efforts have been made to characterize RAD52 structurally and functionally and to better understand its mechanism of action. Nowadays, RAD52 is a validated target for novel synthetic lethality strategies, and its potential in overcoming drug resistance and sensitizing anticancer therapies has been corroborated^22^.

Full-length RAD52 (FL-RAD52) is a protein of 418 amino acids, but it is not stable in its monomeric form. FL-RAD52 physiological form is promoted by the association of the N-terminal portions (212 amino acids each) of multiple FL-RAD52 molecules arranged in a ring-shaped structure on which DNA wraps around^2,23,24^. The C-terminal domains of the associated monomeric units, whose structural organization has been elusive to date, contain two binding sites for DNA repair protein RAD51 homolog 1 (RAD51) and replication protein A (RPA)^23–25^. For many years, a heptameric ring conformation was ascribed to the associated N-terminal portion of FL-RAD52 based on indirect structural evidence^26–28^. In contrast, X-ray data show that the isolated N-terminal-truncated form of the protein (1–212 aa) is arranged in ring-shaped undecamers characterized by a deep positive charged cleft to accommodate DNA molecules that wrap around the protein ring (inner DNA binding site)^2,24,29^. Notably, a second DNA binding site (outer DNA binding site) was also reported for the N-terminal truncated form of the protein^29,30^. Only recently, a Cryo-Electron microscopy (cryo-EM) structure (3.5 Å) from Kagawa and colleagues showed that also FL-RAD52 forms undecamers^31^. In addition, high molecular weight (mw) superstructures have been associated with FL-RAD52 since each ring unit tends to interact with other protein functional units (RAD52 rings) in a stacked- or side-by-side fashion. The tendency to form these high mw superstructures increases in the presence of DNA^24,25,32,33^. The complexity of this protein, which is closely related to its physiological role, makes RAD52 a fascinating but also challenging target to study and address from a drug discovery perspective. Indeed, despite the growing interest that this complex target recently attracts due to its oncogenic role in mediating many DDR pathways on which cancer cells rely when canonical ones are impaired, there are no FDA-approved RAD52 inhibitors on the market, and no RAD52 inhibitors are reported to be in clinical development yet (ClinicalTrials.gov)^22^. This is a serious shortcoming/weakness, as it hinders the possibility of exploiting a validated target that may significantly enhance SL therapies by overcoming drug resistance and side effects^11,15^.

Through a multidisciplinary approach, cryo-EM, Size-Exclusion Chromatography-Small Angle X-ray Scattering (SEC-SAXS), computational simulations, and biophysical analyses, in this work, we confirmed that FL-RAD52 is arranged in undecameric rings (11 monomers each), matching the N-terminal domain organization rather than the previously described heptameric arrangement^31^. This work presents a cryo-EM structure of FL-RAD52 (2.2 Å) at the highest resolution available compared to the structures obtained through X-ray crystallography (N-terminal truncated RAD52 undecameric ring) or cryo-EM (FL-RAD52 undecameric ring) (see Supplementary Table S1). The present data allow the interpretation of the hydration shell of the protein, rarely determined in a cryo-EM structure, and critical for protein stability and function, as well as for prospective drug discovery applications. Indeed, the enthalpic and entropic balance of the change in the hydration shell that takes place upon water molecule displacement by a ligand is critical for ligand binding and it is propaedeutic for understanding the final water molecule rearrangement on the protein-ligand complex (solvation and desolvation)^34^. The data presented here provides a detailed knowledge of the water molecules in the hydration shell of FL-RAD52 that can be critically exploited to predict and improve the binding affinity of potential ligand molecules for the target.

Furthermore, with our analyses, we corroborate and describe through simulation and modeling the extreme flexibility of the FL-RAD52 C-terminal domain, which, as in its recombinant form, is an intrinsically disordered portion of the full-length protein. This observation further draws attention to the hitherto minor role played by the FL-RAD52 C-terminal portion of the protein, which may assume a well-defined structure only upon partner binding.

The integrated effort presented here to study FL-RAD52, a promising target in anticancer therapy, provides critical information to promote and support new approaches to drug discovery and paves the way for new insights into its mechanism of action.

## Results

### The 2.2 Å FL-RAD52 protein, as revealed by cryo-EM

**Fig. 1:**
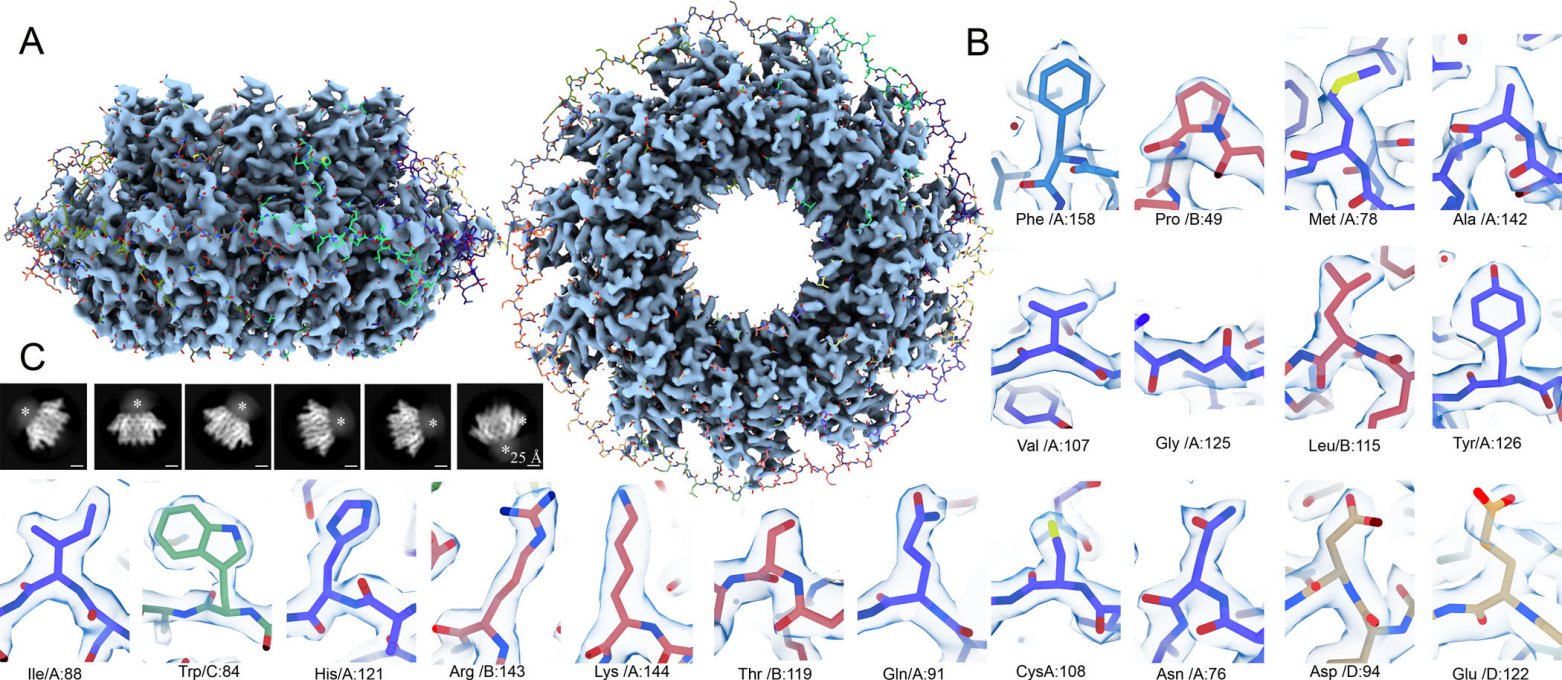
Cryo-EM structure of the FL-RAD52. **A** Human RAD52-FL cryo-EM density map resolved at 2.2 Å average resolution fitted with the model (PDB ID: 8BJM) derived from the crystal structure of the RAD52 N-terminal truncated crystallographic model (PDB ID: 1KN0^24^). Both the map and the model are visible in side- (left) and top- (right) views. The volume is shown at a high-density threshold (≈5σ). **B** Single-amino acid FL-RAD52 map densities fitted with their respective model. **C** representative 2D class averages showing the RAD52 complex in side views. The asterisks point to an electron-dense undefined cloud at the top of the ring.

Human FL-RAD52 (47 kDa) was expressed and purified as a recombinant protein with a 6xHis tag at the N-terminal (Supplementary Fig. 4). A single particle cryo-EM analysis of this sample was pursued to obtain a comprehensive and detailed structural description of this complex and dynamic target, but also to prompt more effective drug discovery campaigns on FL-RAD52 as there are no 3D structures available with resolutions that would allow such an analysis. In the analysis, all the obtained reference-free top views 2D class averages show a precise c11 symmetry (Supplementary Fig. 5). Despite our extensive data analysis, no minor 2D or 3D classes corresponding to alternative ring organizations were detected (Supplementary Fig. 5, 6). The c11 symmetry was therefore applied, allowing to obtain an electron density map of the FL-RAD52 at 2.2 Å average resolution (0.143 FSC correlation criterion) higher than without imposing any symmetry (2.4 Å and 2.7 Å) (Fig. 1A, B, Supplementary Fig. 5)^35^.

As observed in other structures of RAD52 N-terminal, our model comprises a mushroom-like closed ring composed of a stem region formed by the β-β-β-α fold of each monomer (residues 79–156), and by a domed cap region at both the N- and C-terminal of the stem region, that ends with a flat top (Table 1, Fig. 1A)^24,29^. The stem region is relatively rigid, except for some portions in loops L6 and L7. Instead, in the domed cap, the β-hairpin loop (part of β-sheet β1, the loop L3, and part of β-sheet β2), a large portion of the L10 loop, and small regions at the top of the domed cap, corresponding to the N- and C-terminal portions of FL-RAD52, are somewhat flexible, as pointed out by the ResMap analysis (Supplementary Fig. 5). Most amino acid side chains between serine (Ser) 25 and cysteine (Cys) 208 were well resolved and clearly recognizable (Fig. 1B). Moreover, we could visualize amino-acids valine (Val) 23, leucine (Leu) 24, arginine (Arg) 209 (only in chains B, F, I and K) not present in any other RAD52 published structure (Supplementary Fig. 7). Interestingly, densities at the N- (residues 1–22) and C-terminus (residues 209–430) were largely absent (Fig. 1A). In particular, the large C-terminal region appeared in the majority of the 2D class averages side views as an undefined electron-dense cloud, close to the top of the ring (Fig. 1C, and Supplementary Fig. 5) as also observed in the two best resolved 3D class averages (Supplementary Fig. 6).

**Table 1:** Cryo-EM data collection, refinement and validation statistics

	#1 name (EMDB-16089) (PDB 8BJM)
**Data collection and processing**	
Magnification	165000
Voltage (kV)	300
Electron exposure (e–/Å^2^)	50
Defocus range (μm)	−0.8 um to −1.8
Pixel size (Å)	0.731
Symmetry imposed	C11
Initial particle images (no.)	2325722
Final particle images (no.)	837272
Map resolution (Å) FSC threshold	2.2 0.143
Map resolution range (Å)	1.6−3.5
**Refinement**	
Initial model used (PDB code)	1KN0
Model resolution (Å) FSC threshold	2.2 0.143
Model resolution range (Å)	2.49−2.18
Map sharpening *B* factor (Å^2^)	−66
Model composition Non-hydrogen atoms Protein residues Ligands	16655 2050 H_2_O
*B* factors (Å^2^) Protein Ligand	48.97 45.72
R.m.s. deviations Bond lengths (Å) Bond angles (°)	0.33 0.50
Validation MolProbity score Clashscore Poor rotamers (%)	1.31 5 1.17
Ramachandran plot Favored (%) Allowed (%) Disallowed (%)	100 0 0

### FL-RAD52 hydration shell and the H-bond mediated protomer interaction

**Fig. 2:**
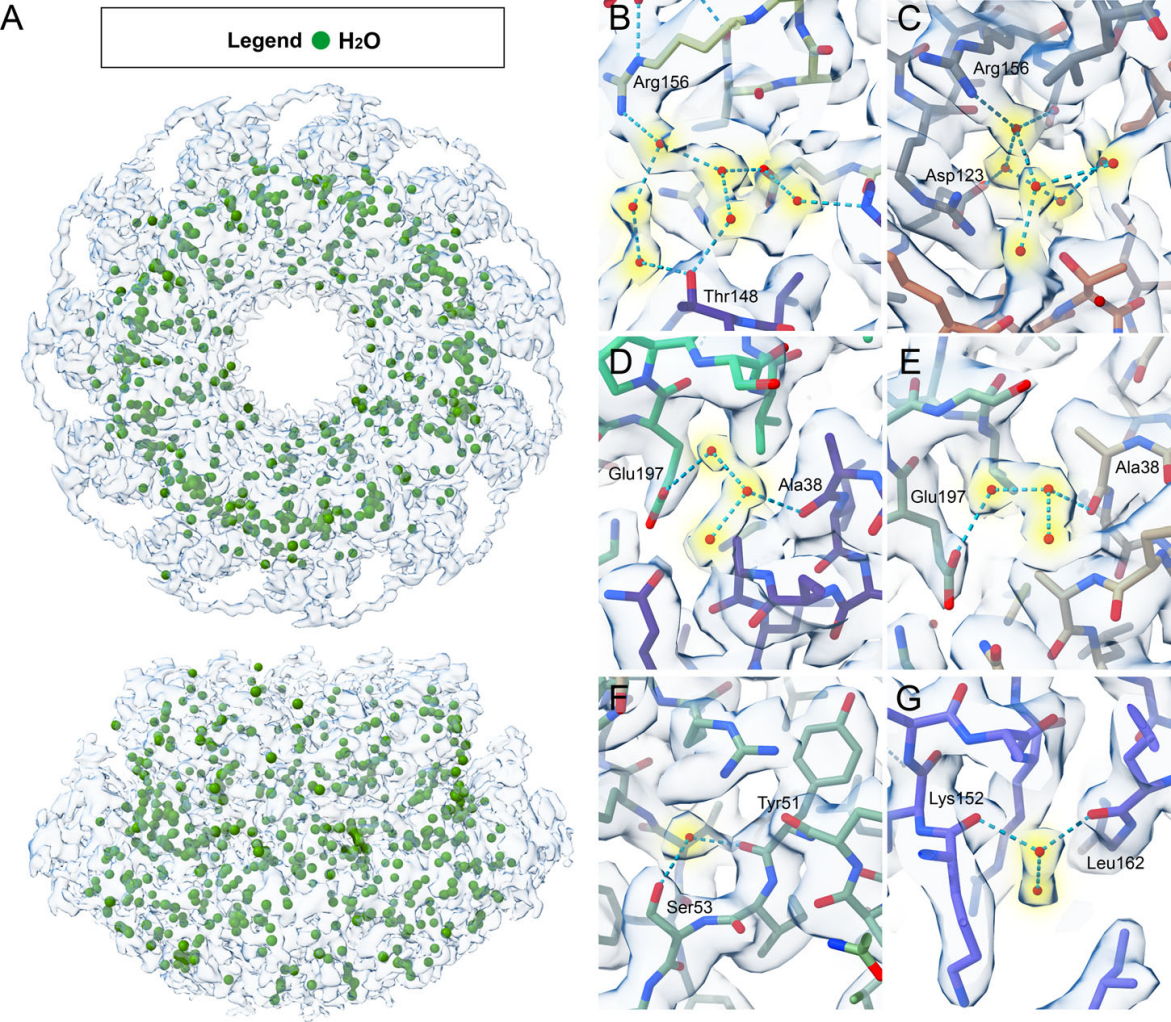
Human FL-RAD52 hydration shell. **A** Hydration shell (green spheres correspond to water molecules) of the FL-RAD52 in both bottom (top) and side (bottom) views. **B**–**G** Visualization of representative H-bound water molecules in the structure of the protein complex.

**Fig. 3:**
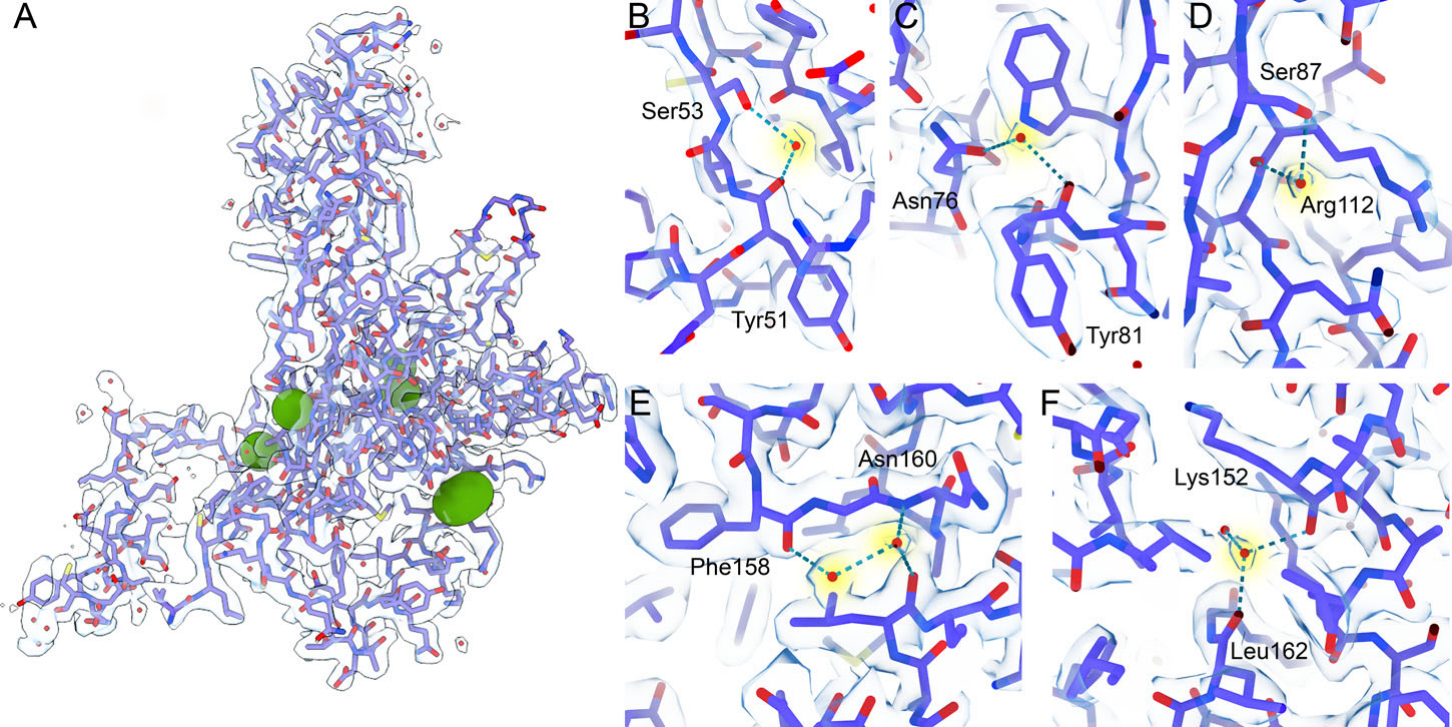
Intra-protomer water-mediated human FL-RAD52 H-bonds. **A** Cryo-EM density map and model (PDB ID: 8BJM) of a representative FL-RAD52 single protomer. The green spheres indicate the water molecules involved in H-bonds observed in most FL-RAD52 protomers. **B**–**F** Details of the intra-protomer water-mediated H-bond interactions are shown in **A**.

**Fig. 4:**
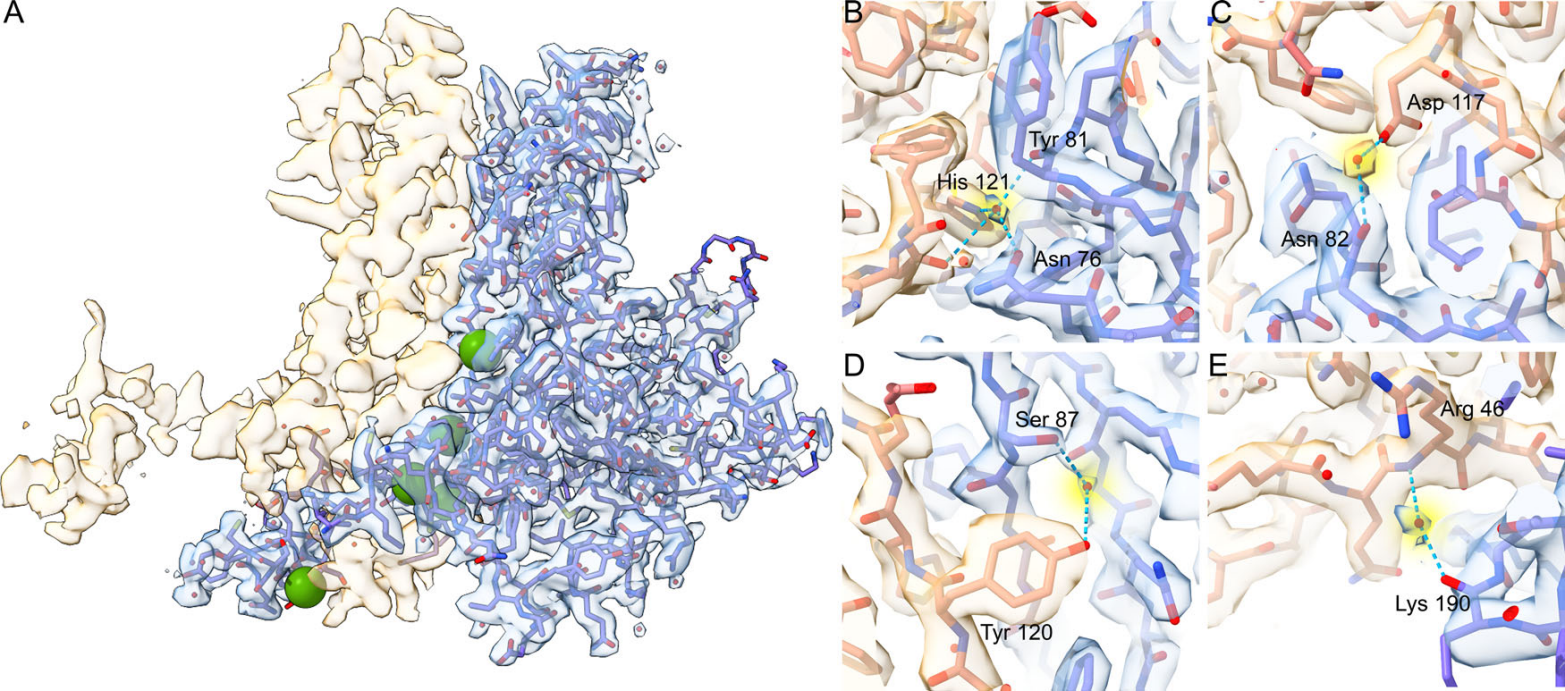
Inter-protomer water-mediated human FL-RAD52 H-bonds. **A** Cryo-EM density maps of two representative adjacent protomers. Only one protomer is fitted with the corresponding FL-RAD52 cryo-EM model (PDB ID: 8BJM). The green spheres indicate the water molecules involved in H-bonds observed between all adjacent protomers. **B**–**E** Details of the water-mediated H-bond interactions observed between all adjacent hRAD52-FL protomers are shown in (**A**).

**Fig. 5:**
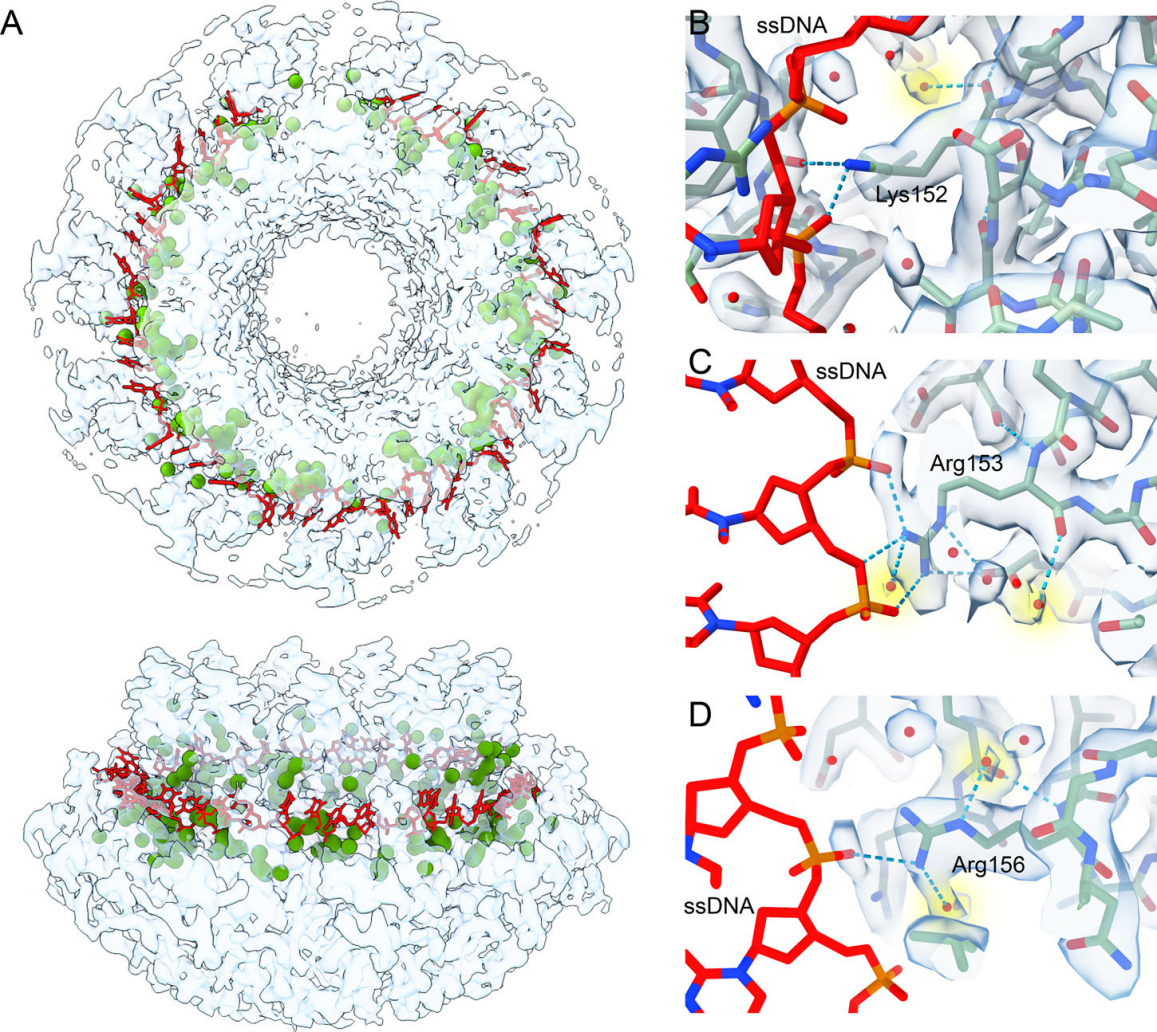
Water molecules at the inner DNA binding site of FL-RAD52. **A** Human FL-RAD52 cryo-EM electron density map in bottom (top) and side (bottom) view showing the water molecules (green spheres) close to the inner ssDNA binding site (from PDB ID: 5XRZ^28^). **B**–**D** Details of the water-mediated H-bond interactions involving respectively Lys 152, Arg 153, and Arg 156 (PDB ID: 8BJM).

The 2.2 Å resolution structure obtained from our cryo-EM analysis enabled a hydration shell analysis that can provide information critical to an accurate understanding of the structure, dynamics, and interaction of the protein with possible partners or cofactors. Densities corresponding to 498 water molecules, equivalent to about 45 water molecules per protomer, were identified in our cryo-EM map (Fig. 2A). These water molecules are connected to the protein ring by hydrogen bonds and coordinate to FL-RAD52 single or multiple polar residues and to the polypeptide backbone, also forming connected chains (Fig. 2B–G). Water molecules mediate hydrogen bond connections inside each protomer and between adjacent protomers (Figs. 3A, 4A and Tables S2, S3). Specifically, in the large majority of protomers, intra protomer water-mediated hydrogen bonds are preserved between serine (Ser) 53 and tyrosine (Tyr) 51, and between asparagine (Asn) 76 and (Tyr) 81, in the domed cap N-terminal region (Fig. 3B, C); between serine (Ser) 87 and arginine (Arg) 112, in the stem region (Fig. 3D); and between phenyl-alanine (Phe) 158 and asparagine (Asn) 160, in the C-terminal region (Fig. 3E). Finally, an additional preserved water-mediated hydrogen bond between lysine (Lys) 152 and leucine (Leu) 162 (Fig. 3F) is linking the protomer domed cap to the stem region. Notably, the H-bond network also mediates the interactions among stem regions of adjacent protomers (Fig. 4 and Table S3). For instance, in all protomers asparagine (Asn) 76, tyrosine (Tyr) 81, asparagine (Asn) 82 and serine (Ser) 87 establish water-mediated hydrogen bonds with histidine (His) 121, aspartate (Asp) 117 and tyrosine (Tyr) 120, respectively (Fig. 4B–D). Water-mediated hydrogen bond interactions also connect the C- with the N-terminal regions of the domed cap of adjacent protomers (i.e, between lysine (Lys) 190 and arginine (Arg) 46 (Fig. 4E)). In summary, this analysis allowed the identification of a water molecules network that stabilizes not only each protomer unit, but also the protomer-protomer interactions, i.e., the overall N-terminal ring structure. In addition, in our 3D structure, several water molecules were also observed at the inner DNA binding site of FL-RAD52 (Fig. 5)^29^. In this region, several key residues, known to electrostatically interact with the phosphate backbone of the ssDNA, namely lysine (Lys) 152, arginine (Arg) 153 and (Arg) 156 are involved in water-mediated hydrogen bonds (Fig. 5B–D, Table S2).

### Comparison with other RAD52 structures: the role of Arg 55

**Fig. 6:**
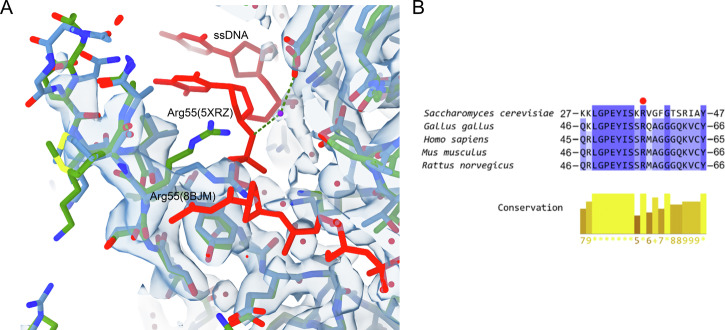
Arg55 role in DNA binding. **A** Cryo-EM map of FL-RAD52 β-hairpin loop portion containing Arg55 (Loop L3) fitted with the obtained cryo-EM model (in light blue, PDB ID: 8BJM) with superimposed the RAD52_25-208_ inner DNA binding site model (in green, PDB ID: 5XRZ^28^). The ssDNA at the inner DNA binding site is in red (**B**) Multiple sequence alignment analysis showing the conservation of Arg55 (highlighted with a red dot) in different species.

The FL-RAD52 model reported here shows a typical undecameric organization in agreement with previously published structures of RAD52^2,24,29^, even though with a higher resolution. The average root-mean-square deviations (RMSD) between the available structures range from 0.5 to 0.6 Å (Table S4), with the largest Cα to Cα distance mainly observed in the Gly (60)-Cα, inside the flexible β-hairpin loop L3. Concerning the per-residue side-chain atom deviations, our structure differs from the published ones in some charged amino acids, mainly located in the flexible domed cap of the protein (Table S5). Among the residues essential for ssDNA binding at the inner binding site^29^, only Arg 55 (hairpin loop, β-sheet β1), highly conserved among different organisms, significantly differs in position from other crystallographic structures (Fig. 6). Interestingly, in our model (PDB ID: 8BJM) Arg55 potentially clashes with the phosphate-deoxyribose backbone of ssDNA with respect to PDB ID 5XRZ^29^, (RAD52_25-208_ - ssDNA complex) (Fig. 6). Finally, the residues in the outer DNA binding site, involved in the ssDNA interaction, are clearly less defined in our cryo-electron density map (e.g., Lys 102 (β-sheet β4) and Lys 133 (domed cap, loop L8, PDB ID 5XS0^29^, (Supplementary Fig. 8)).

### FL-RAD52 self-oligomerization and DNA binding

The high-resolution cryoEM structure of FL-RAD52 provided unprecedented high-resolution structural information on the RAD52 ring structure. Nevertheless, it was still unable to provide similar details for the C-terminal portion, hampering a complete understanding of its organization and function. To better grasp further insights on RAD52 C-terminal domain we compared the FL-RAD52 recombinant protein with the N-terminal truncated form (1–212 amino acids). The two proteins were characterized by similar melting temperatures even though the steaper slope of the melting curve for the full-length protein may suggest varied cooperativity in the melting process (Supplementary Fig. 9). Similar melting temperatures, in agreement with previously reported data on RAD52 thermal unfolding (32), can be ascribed to the unfolding of RAD52 ring structures that characterize both proteins. Indeed, the unfolding curve is recorded as the ellipticity changes at 222 nm as a function of the increasing temperature, i.e., monitoring of the changes in secondary structural elements proper of RAD52 ring. However, Circular Dichroism (CD) spectra deconvolution showed an overall significant increase in the disordered regions of FL-RAD52 compared with RAD52 N-terminal domain (51% and 32%, respectively, Supplementary Fig. 9) in agreement with the purported dynamic nature of the C-terminal portion.The most abundant disordered C-terminal component present in the FL-RAD52 sample, that plays a role in the formation of high molecular weight species that undergo the unfolding process right before the ring structure, is most likely the driving force that leads to a faster and sudden unfolding (steeper unfolding slope) of the structure compared to the truncated protein where the C-terminal region is missing. Despite the increased mobility, the FL protein still retains the ability to bind ssDNA, as proved by fluorescence polarization (FP) assay (Supplementary Fig. 10), which allowed to calculate a *K*_d_ = 37.2 nM similar to the one previously reported for the truncated N-terminal RAD52 (~6 nM). Even if the full-length form retains a significant binding affinity for DNA, the small, but not negligible difference observed between the *K*_d_s could still depend on the role played by the C-terminal in DNA binding. However, further mechanistic and high-resolution analyses are required to conclusively assess the details of the complex DNA binding mechanism to the two proteins. Since DNA binding seems to promote the ability of FL-RAD52 to self-oligomerize in superstructures^24,25,32,33^, further experiments were then pursued to evaluate this behavior in the absence or presence of ssDNA. Initially, MST experiments were set up to probe the protein binding to itself, obtaining an apparent K_d_ for this process of 13 μM (*K*_d_) (Supplementary Fig. 10). In addition, dynamic light scattering (DLS) analyses highlighted the formation of a highly polydisperse and heterogeneous FL-RAD52 sample with the most abundant species having an average hydrodynamic radius of 10.52 nm and an estimated average molecular weight (Mw) of 828.9 kDa, when tested at 17 μM (0.8 mg/mL) (Supplementary Fig. 11). Interestingly, DLS highlighted that also the truncated RAD52 N-terminal domain displayed a similar behavior even though the most abundant species had a smaller average hydrodynamic radius and Mw (6.77 nm, 295.5 kDa) (Supplementary Fig. 11). To further support these results, we also carried out Mass Photometry experiments at 800 nM on both RAD52 FL and truncated samples which confirmed the ability of RAD52 to oligomerize forming superstructures composed of two undecameric units (Supplementary Fig. 12). Overall, these data suggested that the unstructured region of FL-RAD52 could promote the formation of high molecular weight species, most likely constituted by multiple protein rings. To gain a qualitative but representative understanding of the FL-RAD52 self-oligomerization behavior, we also performed 3D reconstruction AFM experiments (Supplementary Fig. 13). The micrographs of FL-RAD52 at 20 nM (1 μg/mL) showed rounded species with a height of 0.57 ± 0.26 nm and a diameter of 15.13 ± 2.16 nm. As FL-RAD52 concentration was increased to 40 nM (2 μg/mL), 80 nM (4 μg/mL), 160 nM (8 μg/mL), reaching 320 nM (16 μg/mL), the protein units maintained their discrete arrangement while increasing in size, compared to conditions of lower protein concentration. The effect induced on protein self-oligomerization by the presence of DNA was then further analyzed. FL-RAD52 particles showed significant changes in morphology in the presence of DNA compared to FL-RAD52 alone, colocalizing on the nucleic acid, and significantly increasing in size (Supplementary Fig. 13). These data are in agreement with previous evidence reporting that the protein retains a physiological tendency in co-localizing on DNA forming superstructures and suggesting a pivotal role for the unstructured C-terminal region of the FL protein in driving the oligomerization process.

### SEC-SAXS reveals that the C-terminal domain of FL-RAD52 is largely flexible and unstructured

**Fig. 7:**
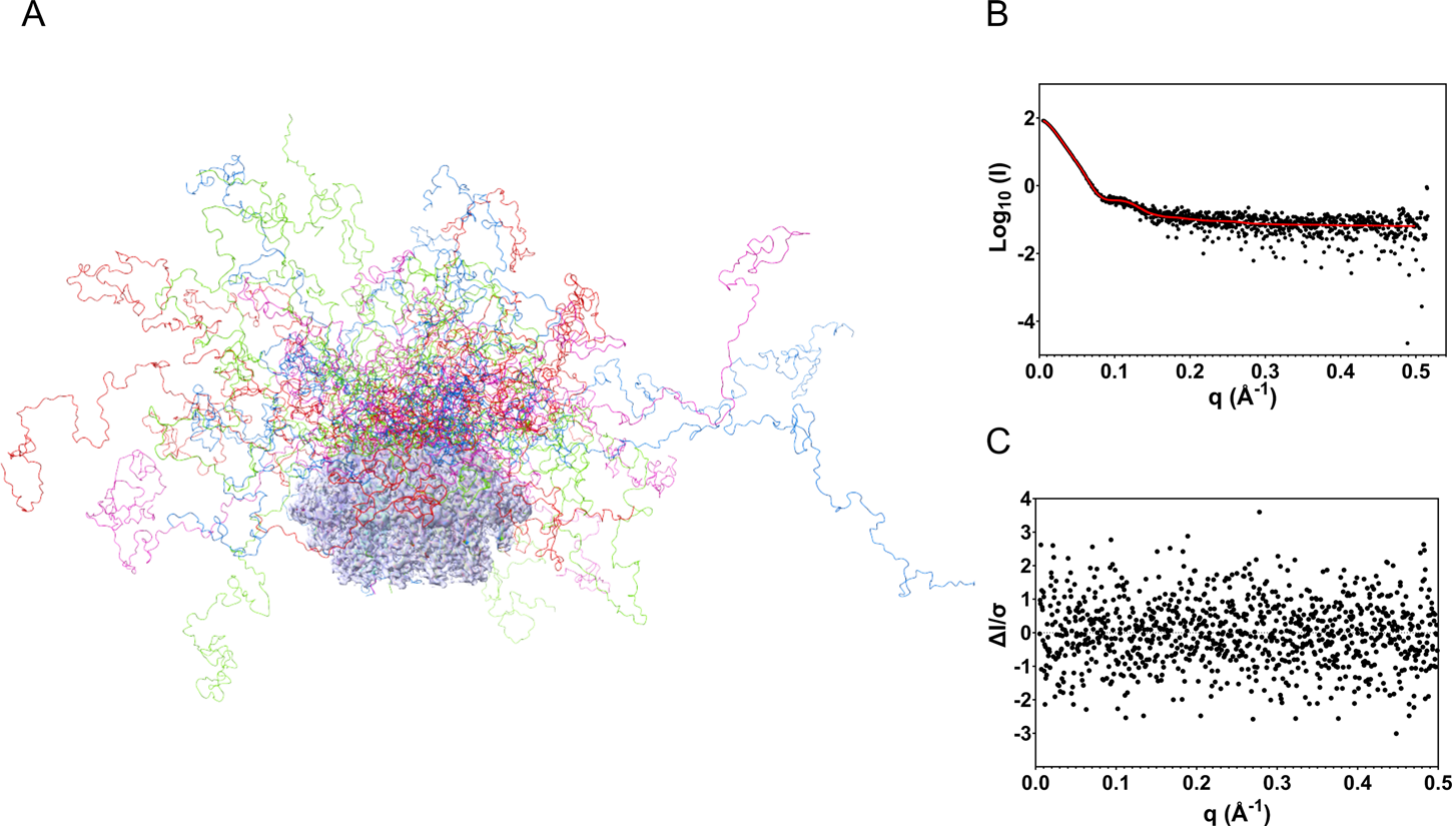
SAXS Modelling of RAD52-FL. ** A** In different colors the overlay of the four most representative SAXS models obtained by modeling the N- terminal and C-terminal FL-RAD52 domains with EOM and utilizing the Cryo-EM structure as rigid body. **B** Model fit to experimental SAXS data. **C** Model residuals.

Given the prominent role that RAD52 C-terminal appears to have on protein function, further characterizations of the dynamic behavior of this protein region were pursued by SEC-SAXS. The primary data analysis showed a molecular weight of roughly 600 kDa (Supplementary Fig. 14, Table S6) and allowed to calculate a radius of gyration (R_g_) of 81.28 Å (Supplementary Fig. 14). Dimensionless Kratky plot confirmed that the protein was folded but partially behaving as a flexible system, as confirmed by the plateau at high q values not decreasing to 0, in agreement with CD and Cryo-EM results (Supplementary Fig. 3, 5)^36,37^. Also, the p(r) function, showed a smooth decrement to 0 and large Dmax of the analyzed particles indicative of protein harboring flexible domains (Supplementary Fig. 14)^38,39^. SAXS data were finally used to carry out atomistic modeling. Initially, the fitting of the obtained Cryo-EM structure was attempted with FoXS. As expected, the lack of flexible domains in this structure yielded a very high χ2, confirming that it was not representative of the experimental data (Supplementary Fig. 15). To compensate for the missing regions, a complete RAD52 undecamer comprehensive of the His-tags and the C-terminal domain was simulated through AlphaFold 2.3 and Alphafold3 (AF2.3, AF3; Supplementary Figs. 16, 17, 18, 19)^40^. Interestingly, AF2.3 predicted the RAD52 C-terminal domain as an unstructured region, in line with previous experimental evidence of our work. By contrast, AF3 predicted, with low confidence, a long portion of it as an alpha-helix, which we did not observe in our experimental data. This discrepancy is probably due to AF3’s higher tendency to hallucinate when predicting intrinsically disordered regions. Therefore, we decided to fit model obtained with AF2.3 through FoXS which significantly improved the χ^2^ to 28 (Supplementary Fig. 20). Based on these results, it was decided to exploit the Ensemble Optimization Method to model the disordered regions at the N-terminal (amino-acids 1–36, comprising the His-Tag) and the C-terminal domains (amino-acids 223–432, including the His-Tag)^39,41^. In this case, the modeling excellently fitted the experimental data with a χ^2^ of 0.960, thus allowing to appreciate the extreme flexibility of the C-terminal domain in solution, for the first time (Fig. 7, Supplementary Fig. 21).

## Discussion

To the best of our knowledge, this work presents the first comprehensive description of the 3D structure of FL-RAD52 obtained by integrating biophysical and structural analyses. The Cryo-EM analysis yielded the FL-RAD52 N-terminal ring structure at the highest resolution available (2.2 Å) to date (PDB: 8BJM). The number of cryo-EM structures resolved at 2.0–2.4 Å is less than 5% out of the total cryo-EM structures present in the protein data bank with a molecular weight within +/− 10% of that of the FL-RAD52 undecamer (77 out of 1660 deposited structures). Our extensive single particle analysis unambiguously shows that FL-RAD52 protein forms undecameric ring structures, in agreement with previous crystallographic RAD52 N-terminal domain studies^24^ and with a more recent Cryo-EM work reporting a 3.5 Å resolution structure of the FL protein^31^. In addition, the resolution achieved in our work allowed a detailed description of the protein hydration shell, providing an unprecedented and indispensable tool to understand the protein interaction with DNA and to pursue drug discovery studies. Indeed, water molecules are not merely part of the fluid the protein emerges into, but they play a key role in protein folding, stability, dynamics, and function^42,43^. In the hydration shell analysis of FL-RAD52, the coordinated water molecules are observed to form a network of hydrogen bonds, inter and intra-protomers, leading to the peculiar structural ring stability, which is required for correct DNA binding. Actually, the presence of water molecules observed in the highly polar FL-RAD52 inner binding site is typical of DNA-binding proteins that are characterized by highly solvated protein-DNA interfaces^42^. The observed water-mediated hydrogen bonds directly involve protein residues critical for ssDNA binding (inner binding site), suggesting a role of water molecules in protein-DNA recognition^42^. When DNA binds the target protein, it must displace the water molecules from the protein surface and energetically compensate for the lost interactions^44^. From the drug discovery perspective targeting the DNA binding pocket, precise mapping of the water molecule network is a valuable tool for predicting the thermodynamic advantage of replacing specific water molecules located within RAD52 pockets with a synthetic ligand. Indeed, it is the thermodynamic change that takes place upon desolvation, when the water molecules are displaced from the binding pocket together with the water molecules rearrangement and the new network formed upon complex formation, which is the driving force for ligand binding^45^. The advantage of an accurate knowledge of the protein hydration shell can thus become critical for designing more potent and selective protein inhibitors. The same is true not only for the DNA binding site, but for any RAD52 binding pocket. The accurate knowledge of the arrangement of water molecules in the ring structure of RAD52, provided here, may support the design of protein inhibitors and facilitate the identification of novel hotspots for drug discovery. Indeed, as already reported^22^, inhibition of RAD52 can be achieved directly by targeting the mechanism underlying protein activity, for instance, hampering the binding of critical interactors such as DNA, or indirectly by undermining protein stability, as weakening the protomer-protomer interaction, thus affecting RAD52 ring structure. Albeit being an indirect and challenging approach, targeting protomer-protomer stability may lead to identifying more selective RAD52 inhibitors than the more classical approach of targeting the promiscuous DNA-binding pocket. However, only a few attempts in this direction have been published^22^, hampered by the difficulties of targeting a protein-protein interface. The information provided here can certainly impact this approach, providing critical information on the water network at the protomer-protomer interface and paving the way for a more robust therapeutic approach. Besides this unprecedented analysis of the RAD52 hydration shell, rarely obtained from a cryo-EM analysis, our high-resolution 3D structure also allowed interesting inferences about the protein’s mechanism of action. For instance, Arg55 is essential since it anchors the ssDNA to the FL-RAD52 inner DNA binding site^29^. Once the ssDNA is bound to the inner DNA binding site, Arg55 has been proposed to act uniquely as an entry/exit gate for ssDNA during homology searches^29^. The position of Arg55 in our model is clearly not compatible with the binding of the ssDNA to the inner DNA binding site, suggesting that this residue may also work as an entry/exit gate for ssDNA in the first phases of FL-RAD52-ssDNA interaction, not only during homology search. Indeed, mutation of Arg55 in the truncated form of the protein is reported to lead to defective ssDNA binding, while binding to dsDNA is, in this mutant, almost fully preserved further supporting the role of this residue specifically in ssDNA binding^24^. A cooperative interaction of the inner and outer DNA binding sites with the ssDNA has also been proved for the truncated protein^29^. Assuming that the DNA first binds RAD52 outer binding site, we can speculate that this initial binding may trigger a shift of the Arg55 position, leading to the successive binding to the inner binding site in a concerted mechanism. The flexibility observed for the residues involved in the ssDNA interaction at the outer DNA binding site (Supplementary Fig. 8, less-defined cryo-EM map) supports this hypothesis, as conformational changes in this region are probably needed to allow ssDNA binding. While the single-particle cryo-EM analysis allowed high-resolution information on the largely structured RAD52 N-terminal domain, its C-terminal region appeared as an undefined electron density cloud in 2D and 3D classifications. Only the integration of simulations and SAXS modeling allowed us to grasp additional details on the extreme flexibility of this region and describe the C-terminal portion of the protein as an intrinsically disordered region (IDR)^46^. The function of the C-terminal domain of FL-RAD52 has yet to be fully elucidated. It has already been reported that FL-RAD52 C-terminal mediates the interaction with RPA and RAD51-ssDNA complex^1,23,25,28^, supporting the DNA strand homology search and annealing^47,48^. Nevertheless, we have shown that FL-RAD52, even without DNA, exhibits a greater propensity to form high-molecular-weight superstructures relative to the isolated N-terminal counterpart. Indeed, biophysical analyses (MST, DLS) and AFM data suggest that this region could promote the formation of high molecular weight species, particularly in the presence of DNA. Altogether, this evidence suggests a potential role of the C-terminal domain in enhancing the recruitment of RAD52 undecamers on ssDNA^49^. FL-RAD52 is indeed known to associate with RAD51-DNA complexes forming globular structures along RAD51 nucleoprotein filament^25,28^. Noteworthy, the IDRs are often involved specifically in molecular recognition interactions, being able to wrap up and bind several partners simultaneously by structural accommodation at the binding interfaces, with faster rates of association and dissociation^50^. We may thus speculate that the FL-RAD52 C-terminal region, which in the recombinant form is an IDR region, could bind other FL-RAD52 rings, link both RPA-ssDNA and RAD51-ssDNA, and quickly detach from its partners during presynaptic complex assembly^51^. This hypothesis sheds light on the important role of this region in driving the DNA repair activity of RAD52. To corroborate these speculations, a thorough understanding of FL-RAD52 self-association and DNA binding mechanism, in which its real physiological role and value lie, is needed. In this perspective, the comprehensive structural analysis presented in this work lays the foundation for further structural characterization of FL-RAD52 in the presence of other protein interactors or DNA and a better understanding of its physiological mechanism of action related to superstructure formations. A recent preprint paper has shown a low-resolution cryo-EM structure of DNA-bound RAD52 (bioRxiv preprint 10.1101/2023.11.14.566657) that is certainly moving in this direction, providing progressively more information to develop effective drug discovery strategies to tackle this promising target^52^. In the coming years, understanding the protein behavior in the presence of intracellular partners will be also crucial for the development of novel FL-RAD52 inhibitors and, thus, for synthetic lethality-based anticancer therapies where FL-RAD52 has been recognized as a critical target to induce synthetic lethality and overcome PARP inhibitors (PARPi) resistances.

## Methods

### Protein expression and purification

Histidine-tagged FL-RAD52 expression vector (pET15b; Addgene) was transformed in *E. Coli* BL21 (DE3) Rosetta pLysS (Sigma-Aldrich). Freshly grown colonies were resuspended in Luria Broth (LB) buffer containing 100 ug/mL Ampicillin. Bacterial cultures were shaken at 180 rpm at 37 °C to reach an optical density (OD_600_) of 0.8 and were then induced for 4 h using 0.8 mM isopropil-β-D-1-tiogalattopiranoside (IPTG) at 25 °C. Biomasses were finally harvested and put at −80 °C.

Frozen pellets were thawed and resuspended in lysis buffer (25 mM TRIS-HCl pH 7.5, 500 mM NaCl, 5% glycerol, 10 mM Imidazole, Tween20 0.01%, 2 mM DTT, Protease Inhibitors EDTA-free 1× (Roche)). The cell suspension was then lysed on ice by sonication and centrifuged for 1 h at 30000 g. The resulting supernatant was collected and loaded on a HisTrap HP chromatography column (Cytiva) equilibrated with Buffer A (25 mM TRIS-HCl pH 7.5, 500 mM NaCl, 5% glycerol, 10 mM Imidazole, Tween20 0.01%, 2 mM DTT). Washing was performed with 4% and 8% of buffer B (25 mM TRIS-HCl pH 7.5, 500 mM NaCl, 5% glycerol, 0.5 M Imidazole, Tween20 0.01%, 2 mM DTT). Protein fractions eluted at 40% buffer B and were pooled together and loaded on a 5 mL HiTrap Desalting chromatography column (Cytiva) equilibrated with buffer C (25 mM Tris-HCl pH 7.5, 150 mM NaCl, 5% glycerol, 1 mM DTT, 0.005% Tween20). The eluted protein was finally loaded on HiTrap Heparin HP chromatography column (Cytiva) equilibrated with buffer C. FL-RAD52 pure protein eluted using a linear gradient up to 100% buffer D (25 mM Tris-HCl pH 7.5, 1.5 M NaCl, 5% glycerol, 1 mM DTT, 0.005% Tween20) in five CVs. The protein storage buffer was 25 mM Tris-HCl pH 7.5, 250 mM NaCl, 5% glycerol, 1 mM DTT, and 0.005% Tween20. The purified protein was flash-frozen in liquid nitrogen and stored at −80 °C. The protein yield was roughly 20 mg of protein per liter of bacterial culture.

For histidine-tagged RAD52 N-terminal domain (1–212), a similar expression and purification protocol was followed. Briefly, after the transformation and expression procedure as described above, the bacterial pellet was resuspended in a 50 mL volume of lysis buffer (25 mM TRIS-HCl pH 7.5, 500 mM NaCl, 5% glycerol, 10 mM Imidazole, Tween20 0.01%, 2 mM DTT, Protease Inhibitors EDTA-free 1x (Roche). The cell suspension was then lysed on ice by sonication and centrifuged for 1 h at 30,000 *g*. The collected supernatant was loaded on a 5 mL HisTrap HP chromatography column (Cytiva) equilibrated with Buffer A (25 mM TRIS-HCl pH 7.5, 500 mM NaCl, 5%glycerol, 10 mM Imidazole, Tween20 0.01%, 2 mM DTT). Washing steps were performed with a 4%, 8%, and 40% of buffer B (25 mM TRIS-HCl pH 7.5, 500 mM NaCl, 5%glycerol, 0.5 M Imidazole, Tween20 0.01%, 2 mM DTT). Fractions eluted at 100% buffer B (0.5 M Imidazole) were pooled in the following storage buffer: 25 mM Tris-HCl pH 7.5, 500 mM NaCl, 5% glycerol, 1 mM DTT, 0.005% Tween20. The purified protein was flash-frozen in liquid nitrogen and stored at −80 °C. The protein yield was roughly 36 mg of protein per liter of bacterial culture.

### Circular Dichroism (CD)

Far-UV CD spectra were recorded on a Jasco J-1100 spectropolarimeter (Jasco, Essex, United Kingdom), equipped with a temperature control system, using a 1 mm quartz cell. The spectra were recorded in the far-UV range 190–260 nm, using FL-RAD52 and RAD52 N-terminal protein at 5 μM and 10 μM concentrations, respectively. Assay buffers were optimized to remove CD interference signals, avoiding the use of chlorine ions and glycerol, and maintaining an ionic strength comparable with that of the storage buffers. FL-RAD52 assay buffer was 25 mM phosphate buffer NaPi, pH 7.5 and 250 mM NaF; RAD52 N-terminal assay buffer was 25 mM phosphate buffer NaPi, pH 7.5 and 500 mM NaF. Constant Nitrogen flush at 4.0 L/min was applied. Raw spectra were corrected for buffer contributions, and the detected signal was expressed as mean residue molar ellipticity [*θ*] (deg × cm^2 ^× dmol^–1^).

For protein secondary structure analysis, the scanning speed was set to 100 nm/min, digital integration time to 1 s, and the temperature set to 20 °C for all experiments. Each spectrum was obtained as an average of ten scans. No shaking was applied to the samples during measurements. Data were analyzed using Dichroweb^53,54^. For protein thermal stability analysis, CD experiments were performed using a temperature scan from 20 °C to 95 °C at the heating rate of 1 °C/min. No shaking was applied during data collection. Thermal stability was measured by monitoring the CD signal at 222 nm wavelength during the temperature scan.

### Dynamic light scattering (DLS)

FL-RAD52 and RAD52 N-terminal protein samples were analyzed through DLS to test oligomerization propensity. Specifically, FL-RAD52 and RAD52 N-terminal were tested, immediately after protein purification, in their storage buffers (25 mM Tris-HCl pH 7.5, 250 mM NaCl, 5% glycerol, 1 mM DTT, 0.005% Tween20, and 25 mM Tris-HCl pH 7.5, 500 mM NaCl, 5% glycerol, 1 mM DTT, 5 mM Imidazole, respectively) at 25 °C, at 17 μM (0.8 mg/mL) (FL-RAD52) and 28 μM (0.7 mg/mL) (RAD52 N-terminal). Sizes of the samples were analyzed using Zetasizer Nanoparticles Analyzer Software (Malvern) using the standard operating procedures for size measurements, repeating the measurements scans 13 times for each sample. Collected data were expressed in terms of mass distribution. Reported data are the average of >3 independent experiments.

### SDS-Page

SDS-PAGE was performed using precast polyacrylamide gels (NuPAGE 4–12% BisTris Gel, Invitrogen). Different concentrations of protein samples were mixed with 4× Loading buffer (0.25 M Tris-HCl pH 6.8, 8% SDS, 0.3 M DTT, 30% Glycerol, 0.4% Bromphenol Blue) before denaturation at 95 °C for 5 min. After samples loading, precast gels were run in XCell SureLock Mini-Cell Electrophoresis System (Invitrogen) in MOPS SDS running buffer (Invitrogen) with a constant voltage of 120 mA for about 90 min. Gel was then stained with Coomassie Blue staining buffer (40% EtOH, 10% Acetic Acid, 0.05% w/v coomassie blue G-250) for 15–30 min and distained with a distaining Buffer (8% acetic acid, 25% EtOH). Protein band images were visualized and quantified using ChemiDoc Imaging System (BioRad).

### Microscale thermophoresis (MST)

MST measurements were performed using Monolith NT.115pico instrument (NanoTemper Technologies, Munich, Germany). Assays were conducted at 5–10% (RED dye) LED excitation power and MST power of 40%. Premium capillaries from NanoTemper Technologies were used. Measurements were performed at 25 °C in the following buffer: 25 mM Hepes pH 7.5, 5% glycerol, 250 mM NaCl, 0.05% Tween 20. The recombinant FL-RAD52 protein was labeled with the Monolith labeling kit RED-NHS (ammine dye NT-647-NHS) according to manufacturer indications (NanoTemper Technologies). Before MST experiments, the labeled protein stocks were centrifuged at 20,000 *g* for 10 min to remove aggregates. The affinity parameter *K*_d_ was determined by performing the experiment in parallel on 16 capillaries, each containing a constant concentration of the labeled target (FL-RAD52, 10 nM) and increasing concentrations of unlabeled ligand (unlabeled FL-RAD52, 0.7 nM–25 μM). The recorded MST data were then plotted as Δ*F*_norm_ against the ligand concentration to yield dose-response curves. Experiments were analyzed with MO. Control and MO.Affinity analysis software (NanoTemper Technologies).

### Fluorescensce polarization (FP)

For Fluorescence Polarization (FP) experiments, 10 nM 6-(fluorescein)-conjugated dT_30_ ssDNA (6FAM) (Merck) was added to 16 different concentrations of FL-RAD52 ranging from 0.2 nM to 6.25 μM in assay buffer (25 mM Tris-HCl pH 7.5, 62.5 mM NaCl, 5% glycerol, 1 mM DTT). 100 μL of each reaction was incubated in a flat black 96-plate (Corning) at room temperature for 15 min. FP of samples was then measured using a Spark Microplate multimodal reader instrument (Tecan) with the following setup Excitation wavelength 485 nm; Emission wavelength 535 nm; Emission Bandwidth 20 nm; Integration time 20 μs; reference millipolar (mP) value set at 20 mP. FP values were plotted as a function of protein concentrations and fitted using a sigmoidal dose-response curve with GraphPad Prism 7.0. Data are the average of multiple (>3) independent experiments.

### Mass photometry

Mass photometry measurements were carried out on FL-RAD52 and RAD52 N-terminal protein samples using a Refeyn TwoMP mass photometer (Refeyn Ltd). Data collection were performed for 60 s using AcquireMP software and analyzed using DiscoverMP software, with default settings. Sample measurements were performed by adding 2 μL of protein solution (8 µM) to an 18 μL droplet of 0.22 μm filtered buffers (RAD52 FL 25 mM Tris-HCl pH 7.5, 250 mM NaCl, 5% glycerol, RAD52 N-term 25 mM Tris-HCl pH 7.5, 500 mM NaCl, 5% glycerol). A standard curve generated with bovine serine albumin (BSA) was utilized to infer molecule weights.

### Sequence alignment

Saccharomyces cerevisiae, Gallus gallus, Homo Sapiens, Mus musculus, Rattus norvegicus (Uniprot entries: P06778, P39022, P43351, P43352, D3ZZL1), RAD52 WT amino acidic sequences were retrieved from Uniprot. Sequence alignments were performed with Clustal Ω and edited using Jalview^36^.

### Atomic force microscopy (AFM)

FL-RAD52 samples were diluted in AFM buffer (30 mM MOPS pH 7.3, 3 mM MgCl_2_, 20 mM NaCl) (final concentrations from 20 nM (1 μg/mL) to 320 nM (16 μg/mL)) in the presence or absence of ss (ΦX174 bacteriophage)—or ds (pBR322 plasmid) – DNA (New England Biolabs, USA). 40 nM protein was tested in the presence of 7.5 pM pBR322 dsDNA and also in the presence 80 nM 5 kb-long φX174 viral (+) ssDNA^55^ 20 μL of each sample was loaded onto freshly cleaved mica and left at room temperature for 5 min to allow samples to be deposited on the mica slide. The liquid excess was dried off from the mica and rinsed with a gentle flux of filtered Milli-Q water (Millipore).

Morphological images of protein and protein-DNA complexes were acquired by using a Nanowizard III (JPK Instruments, Germany) mounted on an Axio Observer D1 (Carl Zeiss, Germany) inverted optical microscope. Image data were acquired operating in peak force tapping mode using RTESPA-300 cantilevers (TESPA, Bruker, MA, USA) (125-μm nominal length, 40-μm nominal width, nominal spring constants of 40 N/m, and typical resonant frequencies of 300 kHz). The RTESPA-300 probes have an 8 nm nominal tip radius of curvature. All images were acquired as 256 × 256 pixels images with a scan rate between 0.6 and 1.0 Hz. Images were processed using the JPK Data Processing Software. Measurements of particle diameters and heights were performed on 1 µm-scan-size-images. Reported values are the average of >20 measurements on three independent protein samples.

### Negative staining transmission electron microscopy

Negative staining experiments were performed on purified FL-RAD52 2 μM (0.1 mg/mL) (buffer 25 mM TRIS-HCl pH 7.5, 250 mM NaCl, 5% glycerol, 0.005% Tween20). Briefly, a 5 μL drop of the sample was applied to previously plasma-cleaned 400 mesh copper carbon film grids and stained with 1 wt/v % uranyl acetate solution. Data were collected on a JEM-1011 (JEOL) transmission electron microscope (TEM), with a thermionic source (W filament) and maximum acceleration voltage 100 kV equipped with Gatan Orius SC1000 CCD camera (4008 × 2672 active pixels) (Supplementary Fig. 1).

### Cryo-EM sample preparation and data collection

For cryo-EM grid preparation, a 3 µL droplet of 15 μM (0.7 mg/mL) purified FL-RAD52 sample (in the following optimized buffer: 25 mM TRIS-HCl pH 7.5, 150 mM NaCl, 1% glycerol) was plunged frozen in liquid ethane cooled at liquid nitrogen temperature on glow discharged Quantifoil holey TEM grids (Cu, 300 mesh, 1.2/1.3 um) at 100% humidity and 4.5° C. The grids were blotted with filter paper for 5 s using a Vitrobot Mark IV cryo-plunger (Thermo Fisher Scientific). Grid vitrification optimization was performed on a Tecnai F20 (Thermo Fisher Scientific) Schottky field emission gun transmission electron microscope equipped with an automated cryo-box, an Ultrascan 2kx2k CCD detector (Gatan).

Data screening and high-resolution data acquisition were performed at the EMBL Imaging Centre (Heidelberg, Germany) within the iNEXT project ID 15983. Data screening was performed on 831 gain-corrected counting mode movies acquired with a defocus ranging from −1.2 μm to −2.5 μm. Each movie, with a pixel size of 1.154 Å/pix, was composed of 34 frames collected with a total dose of 40.9 e^−^/Å^2^ (1.2 e^−^/Å^2^/movie frame). This preliminary data set was acquired in EFTEM mode at 6.3 e^−^/pix/sec (8.6 s/movie exposure) on a Thermo Fisher Scientific Glacios SelectrisX Falcon4 EC. The high-resolution data set consisted of 17,400 counting mode movies acquired with a defocus ranging from −0.8 μm to 1.8 μm. Each movie, with a pixel size of 0.731 Å/pix, was composed of 735 EER fractions collected with a total dose of 50 e^−^/Å^2^ (1e^−^/Å^2^/ frame). The data set was acquired in EFTEM mode at 8 e^−^/pix/s on a Thermo Fisher Scientific 300 kV cold FEG IC-Krios equipped with SelectrisX energy filter and Falcon4 EC direct electron detector. Data collection for both screening and high-resolution acquisitions was performed using SerialEM software^56^ (Supplementary Fig. 1).

### Single particle image processing and 3D modeling

All data related to Cryo-EM Data collection are reported in Tables S7 and S8. For the screening session motion correction was performed on all 831 movies (frames 2–31) with dose-weighting using the Relion3.1 MotionCor implementation^57^. Contrast transfer function (CTF) correction was performed with CTFFIND 4.1^37^ on all the 831-dose weighted motion-corrected micrographs. About 3900 representative particles were auto-picked from 10 micrographs (391 particles/micrograph) using the Laplacian of Gaussian (LoG) filter. The obtained preliminary low-pass filtered 2D class averages were then used for automated particle picking. This resulted in 580952 particles being extracted and down-sampled for several iterative rounds of 2D classification and selection. A total of 258839 particles from 20 selected 2D classes were subjected to unsupervised 3D classifications (number of classes *K* = 4) using an FL-RAD52 low-resolution initial model obtained from 2D averages with the 3D initial model algorithm implemented in Relion3.1. Similar 3D classes have been obtained using a sphere as an unbiased initial model. The subsets of particles corresponding to the two more represented classes (class 3, 53% and class 4, 32%) corresponding to 221976 particles, after being re-extracted at full resolution, were used for the final refinement. The final FL-RAD52 electron density map was resolved at 3.4 Å by the 0.143 FSC criterion after post-processing, CTF refinement, and polishing.

Motion correction and CTF estimation were performed ‘on the fly’ during data acquisition for the high-resolution data collection. A preliminary manual particle picking was accomplished on about 30 selected micrographs (around 130 particles/micrograph), followed by a 2D classification on around 3600 extracted particles, which was performed to assess the overall sample quality and set up the correct particle box size. LoG filter parameters and box size optimizations were set up on 100 selected micrographs and then applied for particle auto-picking on all 14700 rescaled (1:2) micrographs. This resulted in 4124609 particles (281 particles/micrograph on average) extracted and down-sampled for several iterative rounds of 2D classification and selection. 2325722 particles from 65 selected 2D class averages were subjected to unsupervised 3D classifications (number of classes *K* = 4) using a FL-RAD52 low-resolution initial model obtained from selected 2D class averages with the 3D initial model algorithm as implemented in Relion 4.0^58^. The subsets of particles corresponding to the more represented 3D classes (class 3 and 4 corresponding to 34% and 36% of particles, respectively), were used separately for the final refinements (Supplementary Fig. 2). After CTF refinement and particle polishing, the best-resolved cryo-electron density map, resolved at 2.16 Å by the 0.143 FSC criterion, was obtained from 3D class 4 extracted particles, imposing c11 symmetry (Supplementary Fig. 2, Table S8). Compared with the FL-RAD52 map with c11 symmetry, the map obtained without imposing symmetry was similar although less resolved (Supplementary Fig. 2, 3). The cryo-electron density maps obtained from 3D class 3, corresponding to more than 787000 particles, after CTF refinement and without imposing symmetry, were resolved at 2.6 Å by the 0.143 FSC criterion (Supplementary Figs. 2, 3). The FL-RAD52 protein model (PDB ID: 8BJM) was obtained from the RAD52_25-208_ crystal structure (PDB ID: 1KN0^24^) after several iterative cycles of Phenix real-space refinement^59^ and COOT^60^ manual adjustments. Cross-correlation analyses, measures of distances, model superimposition, 3D visualizations, and rendering were performed using Chimera^61^ and ChimeraX^62^. The comparison between the different RAD52 structures was also performed using 2StrucCompare^63^. To add water molecules, a semi-automatic approach using phenix.douse (Phenix suite^64^) and visual inspection in ChimeraX (CheckWaters) and Coot was applied. The water molecules were validated based on the shape of the local density, map value, and location at the right distance range for hydrogen bonding to the polar group in the vicinity. Models were refined using real-space refinement as implemented in Phenix^65^ and validated using MolProbity^66^.

### Size-exclusion chromatography-small angle X-ray scattering (SEC-SAXS)

SEC-SAXS experiments of FL-RAD52 were performed at the BM29 beamline of the European Synchrotron Radiation Facility (ESRF, Grenoble, France)^67^. 100 µL of the sample were injected into a Superose 6 3.2/300 pre-equilibrated in 20 mM Tris pH 7.5, 250 mM NaCl, 1% glycerol, and the flow set to 0.075 mL/min. Data collection parameters are listed in Table S6^68^. SEC-SAXS data were analyzed using Chromixs^65^. Scattering frames corresponding to samples were selected, averaged, and subtracted from averaged buffer frames. The buffer-subtracted, 1D scattering curves were then processed using BioXTAS RAW to compute the radius of gyration, the dimensionless Kratky Plot, and obtain molecular mass estimates (Table S6)^66^. Data were exported in CSV format and re-graphed using GraphPad Prism 9 software. Considering the elevated number of N-terminal and C-terminal flexible residues to be modeled, it was decided to generate small pools of 2000 models each through iterative cycles of RANCH (RANdom Chain generator) run via ATSAS online and utilizing the solved Cryo-EM structure as free-rigid body^39,41,69^. Input parameters are reported in Table S6.

Once 20000 models were created, these were locally pooled in a unique ensemble, and Form Factor MAKER (FFMAKER) was locally run^39,41,69^. The output file was then utilized for running Genetic Algorithm Judging the Optimization of the Ensemble (GAJOE)^39,41,69^. Model fittings, residuals, and Rg distributions were graphed using Graphpad Prism 9. SAXS data have been deposited into SASBDB with accession number SASDQ49—His-Tagged full-length DNA repair protein RAD52 homolog^70^.

### Alphafold model generation and fitting in SAXS data

A complete FL-RAD52 undecamer (comprehensive of protein tags) was generated with AlphaFold 2.3.2, (model preset = multimer, no amber relaxation) run as a Singularity container on one node of the IIT-HPC Cluster Franklin using as a GPU a NVIDIA Ampere A100. Database preset was set to full database (full_dbs, databases: bfd, mgnify, pdb_mmcif, pdb_seqres, pdb70, uniref30_022023, uniprot, uniref90). Graphs of multiple sequence alignment (MSA) coverage, sequence similarity predicted local distance difference test (pLDDT) and predicted aligned error (PAE) were generated through a Python script adapted from https://raw.githubusercontent.com/jasperzuallaert/VIBFold/main/visualize_alphafold_results.py, https://raw.githubusercontent.com/busrasavas/AFanalysis/main/AFanalysis.py. Alphafold 3 predictions were carried out through AF3 server https://golgi.sandbox.google.com/about. Graph of PAE was generated through Python script adapted from https://github.com/flyark/AFM-LIS. For SAXS modeling, the Cryo-EM structure or the generated AF prediction was used to evaluate model agreement with experimental data through FoXS.

### Statistics and reproducibility

Details on statistical analysis and reproducibility are available in the reporting summary.

### Reporting summary

Further information on research design is available in the Nature Portfolio Reporting Summary linked to this article.

### Supplementary information

Supplementary Information

Reporting Summary

## Data Availability

The structural data presented have been deposited to the PDB with ID: 8BJM and to the SASBDB with ID: SASDQ49.

## Supplementary information

Additional figures and tables, including reports of structural data collection and analyses, are available in an online version containing supplementary material available at 10.1038/s42003-024-06644-1.
