# Supplementary material for: An integrative structural study of the human full-length RAD52 at 2.2 Å resolution
Source: Commun Biol. 2024 Aug 8;7:956. doi: 10.1038/s42003-024-06644-1 (PMC11306251; doi:10.1038/s42003-024-06644-1)
Supplement: Supplementary file 1 — Supplementary Information [file 42003_2024_6644_MOESM1_ESM.pdf]

Supplementary Information

An integrative structural study of the human full-length RAD52 at 2.2 Å resolution

Beatrice Balboni[1,2,#], Roberto Marotta[3,#], Francesco Rinaldi[1,2], Giulia Milordini[1], Giulia Varignani[1,2], Stefania Girotto[1,4]*, Andrea Cavalli[1,5]*

[1]Computational and Chemical Biology, Istituto Italiano di Tecnologia, via Morego 30, 16163, Genoa, Italy
[2]Department of Pharmacy and Biotechnology, University of Bologna, via Belmeloro 6, 40126, Bologna, Italy
[3]Electron Microscopy Facility (EMF), Istituto Italiano di Tecnologia, via Morego 30, 16163, Genoa, Italy
[4]Structural Biophysics Facility, Istituto Italiano di Tecnologia, via Morego 30, 16163, Genoa, Italy
[5]Swiss Federal Institute of Technology Lausanne (EPFL), Switzerland

[#]Contributed equally to this work
*Corresponding and last authors: stefania.girotto@iit.it, andrea.cavalli@iit.it

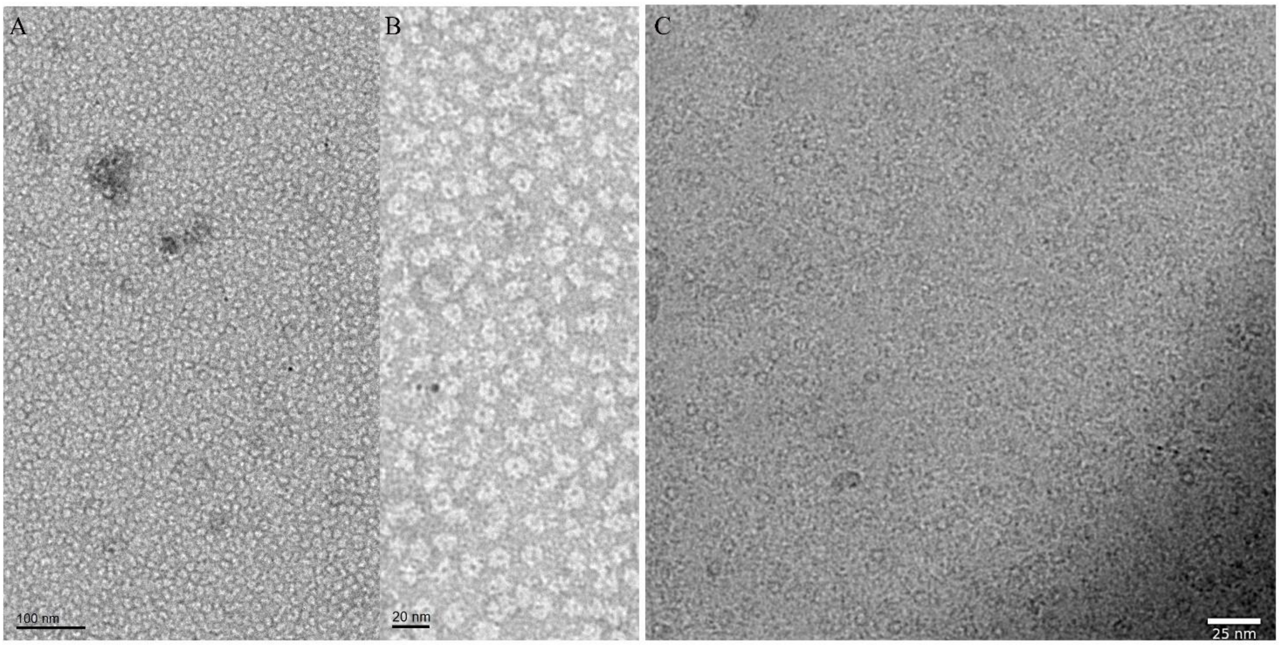

Supplementary Figure 1. Electron microscopy micrographs of FL-RAD52. (A, B) negative stained representative micrographs. (C) cryo-EM representative micrograph.

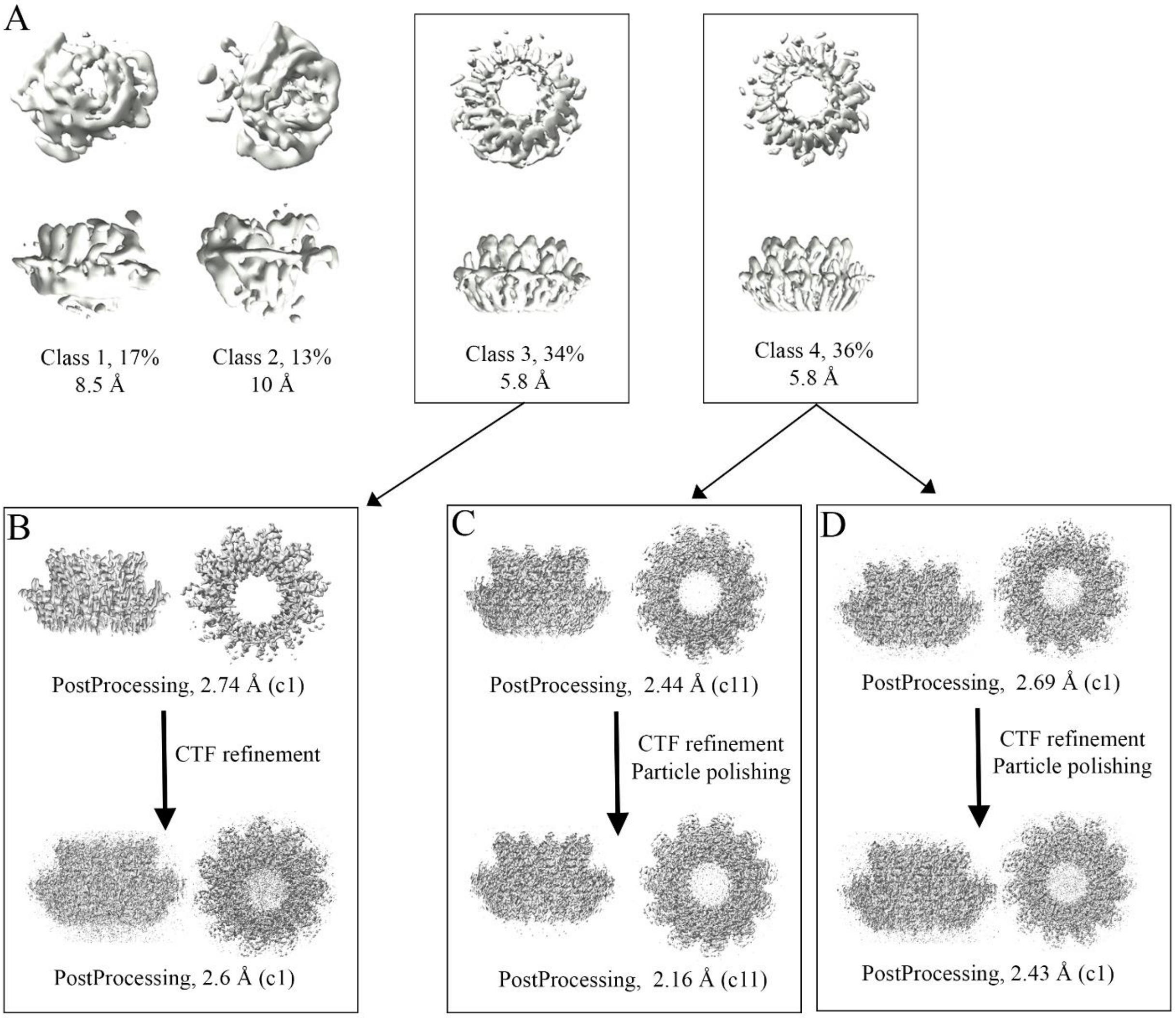

Supplementary Figure 2. FL-RAD52 high-resolution analysis. (A) FL-RAD52 3D class averages in top and side views based on 2325722 particles with their rlnClassDistribution and rlnResolution. (B) FL-RAD52 cryo-EM density maps obtained from 3D class 3 imposing c1 symmetry after CTF refinement. (C) FL-RAD52 cryo-EM density maps obtained from 3D class 4 imposing c11 symmetry after CTF refinement and particle polishing. (D) FL-RAD52 cryo-EM density maps obtained from 3D class 4 imposing no symmetry (c1) after CTF refinement and particle polishing.

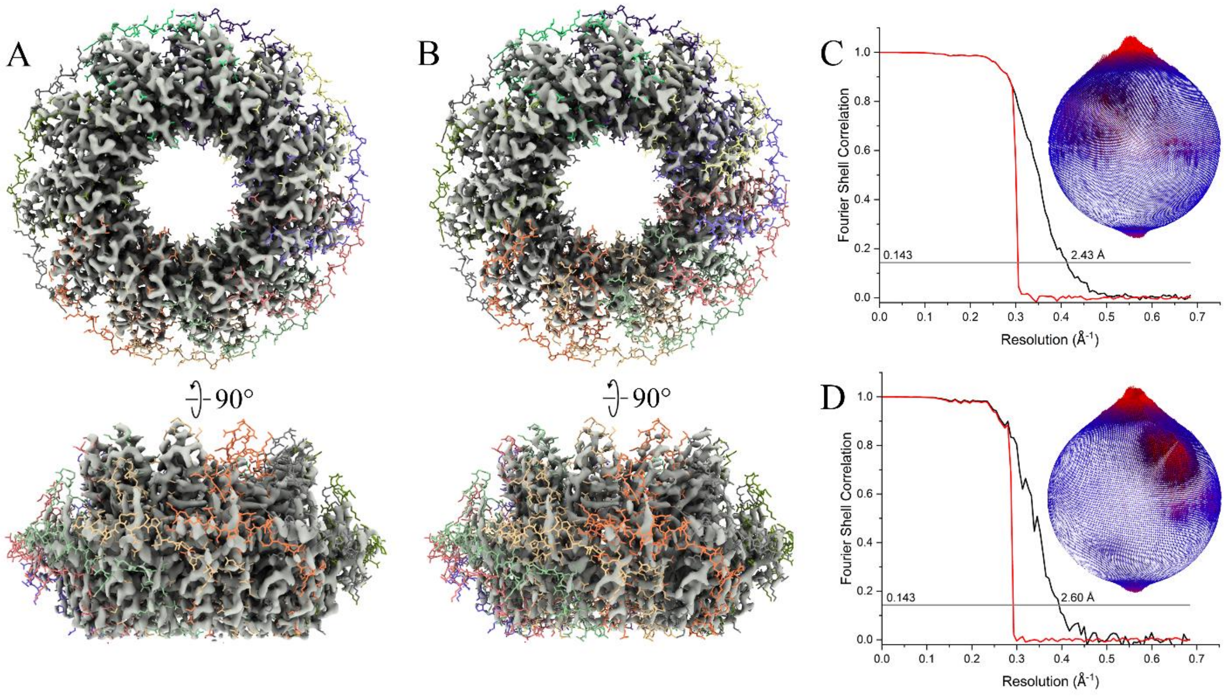

Supplementary Figure 3. (A) FL-RAD52 cryo-EM density maps obtained from 3D class 4 without imposing symmetry (c1) after CTF refinement and particle polishing. (B) FL-RAD52 cryo-EM density maps obtained from 3D class 3 without imposing symmetry (c1) after CTF refinement. (C) FSC curve of the FL-RAD52 of cryo electron density map in A.(D) FSC curve of the FL-RAD52 of cryo electron density map in B.

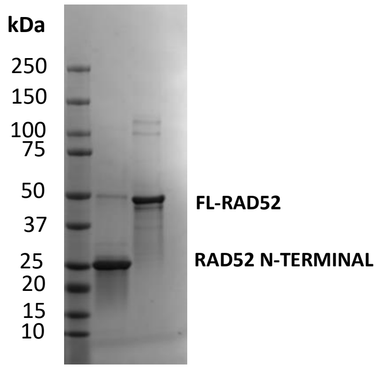

Supplementary Figure 4. Characterization of recombinant FL-RAD52 and N-terminal. SDS-Page analysis of RAD52 N-terminal domain and FL samples.

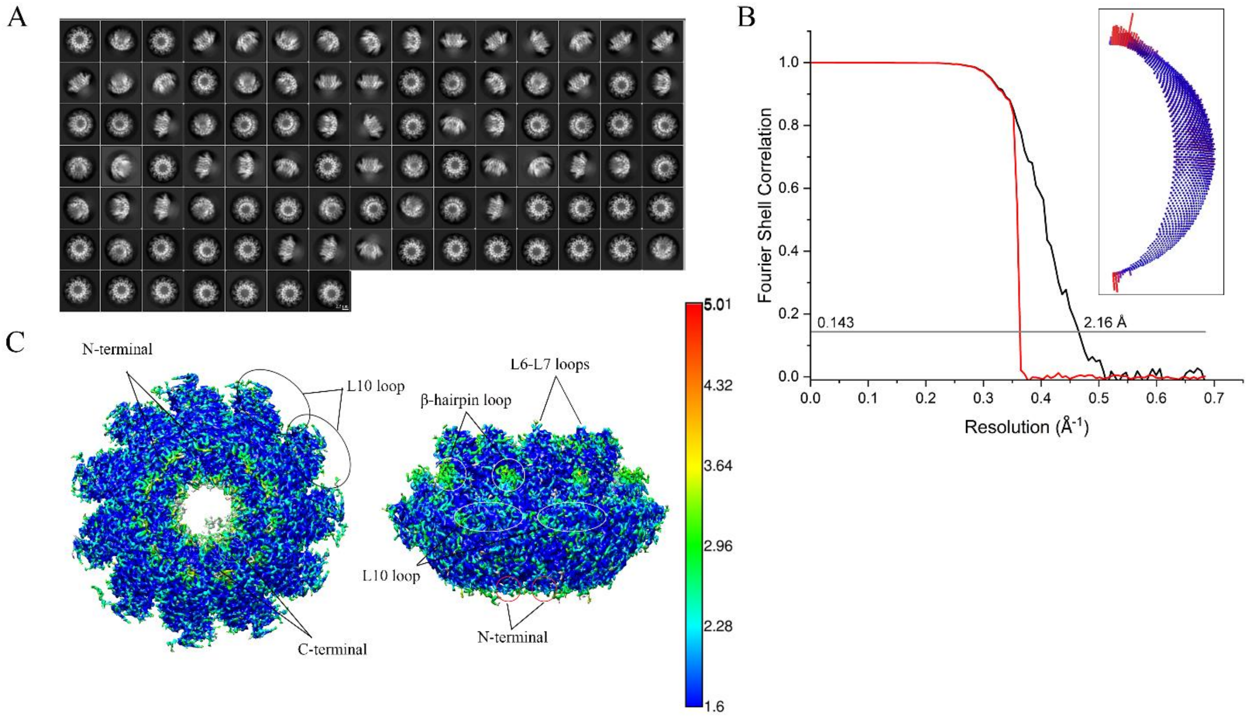

Supplementary Figure 5. (A) representative unsupervised FL-RAD52 2D class averages of 1616139 particles. (B) FSC curve of the FL-RAD52 (red, FSC phase randomized masked curve; black, FSC corrected curve) with a resolution corresponding to FSC=0.143 marked. The inset shows the Euler angle distribution. (C) FL-RAD52 cryo-EM electron density map filtered according to ResMap local resolution.

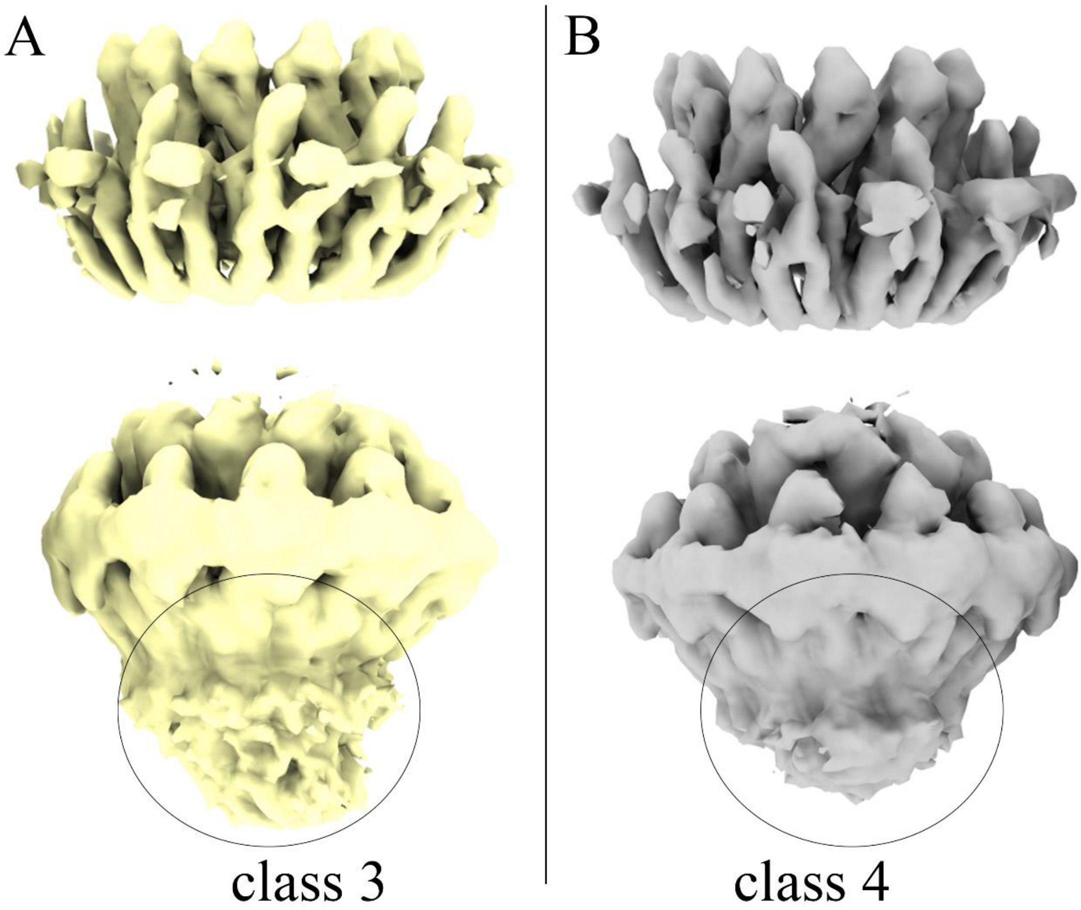

Supplementary Figure 6. (A) Cryo-EM electron density map of FL-RAD52 3D class averages number 3 in side views shown at a higher (up) and lower (bottom) density threshold. (B) Cryo-EM electron density map of FL-RAD52 3D class averages number 4 in side views shown at a higher (up) and lower (bottom) density threshold. Note the presence of an unstructured region close to the top of the ring (encircled).

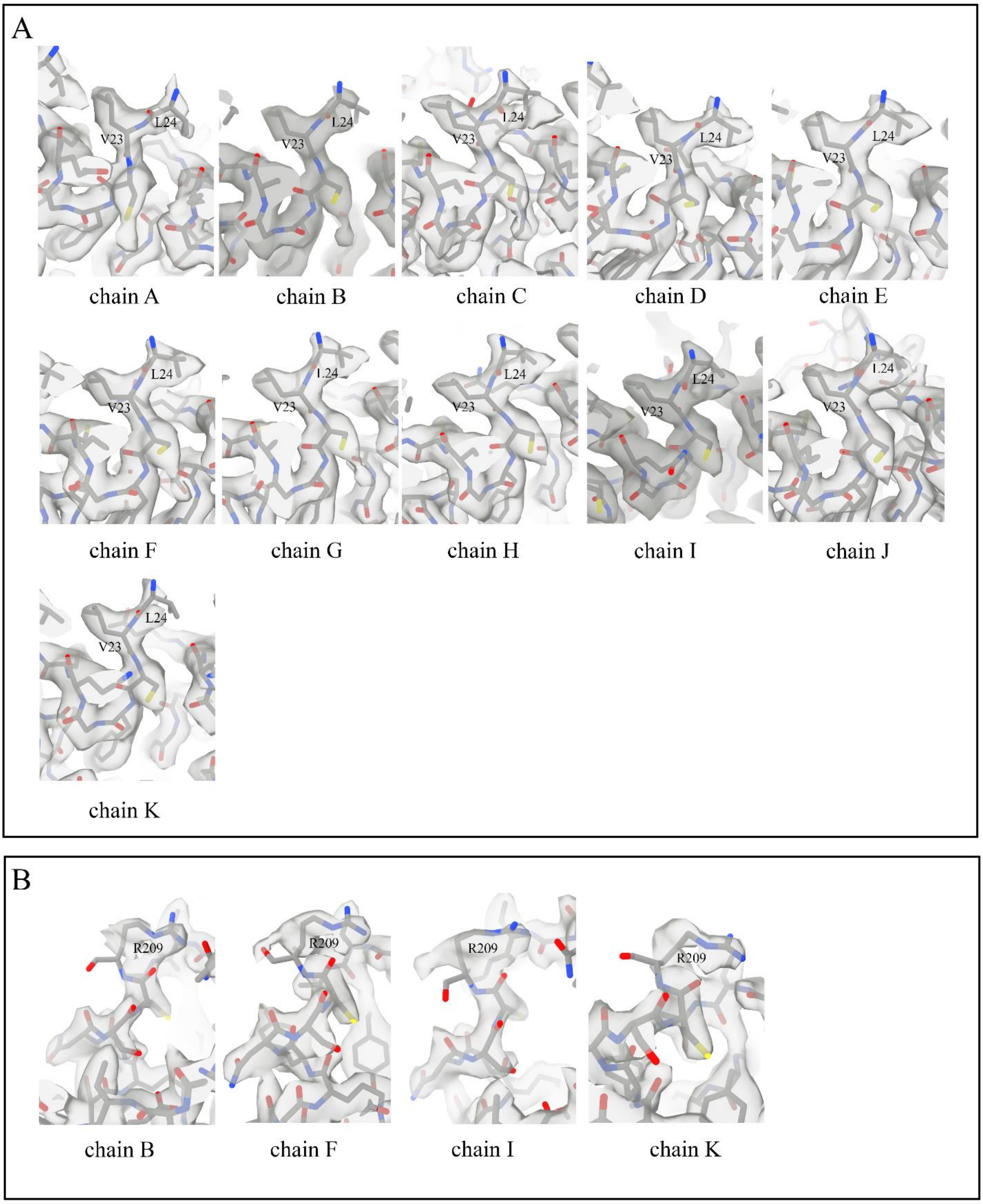

Supplementary Figure 7. Detail of Val 23 and Leu24 residues at the N-terminal (A) and Arg209 residues at the C-terminal (B) of the FL-RAD52 model. The model is fitted in its corresponding cryo-EM electron density map.

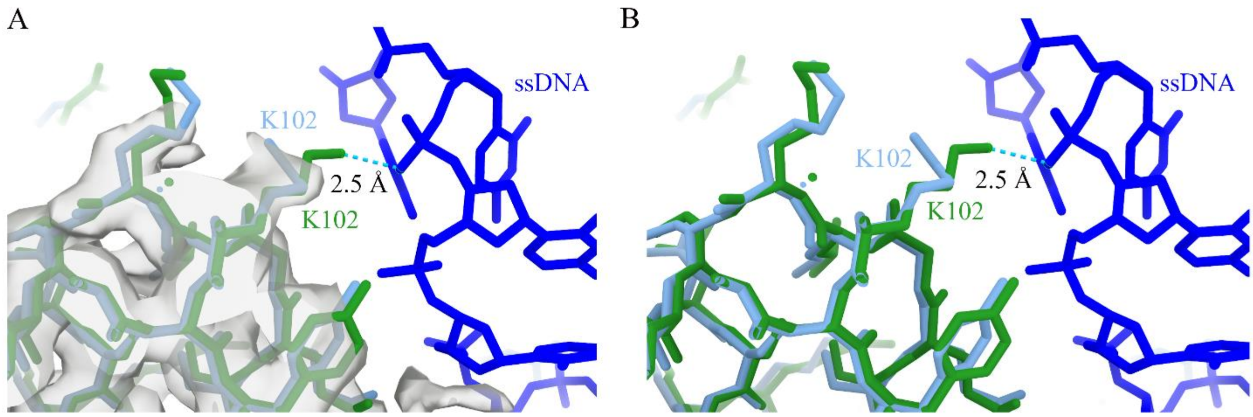

Supplementary Figure 8. Detail of the FL-RAD52 model region containing Lys102 (in cyan) and fitted in the cryo-EM electron density map. The model is superimposed to the same region from the crystal structure of the $RAD52_{25\text{-}208}$ outer DNA binding site model (PDB ID: 5XS0[28]) in green. Note that the cryo-electron density map is shown at low density threshold (≈2σ).

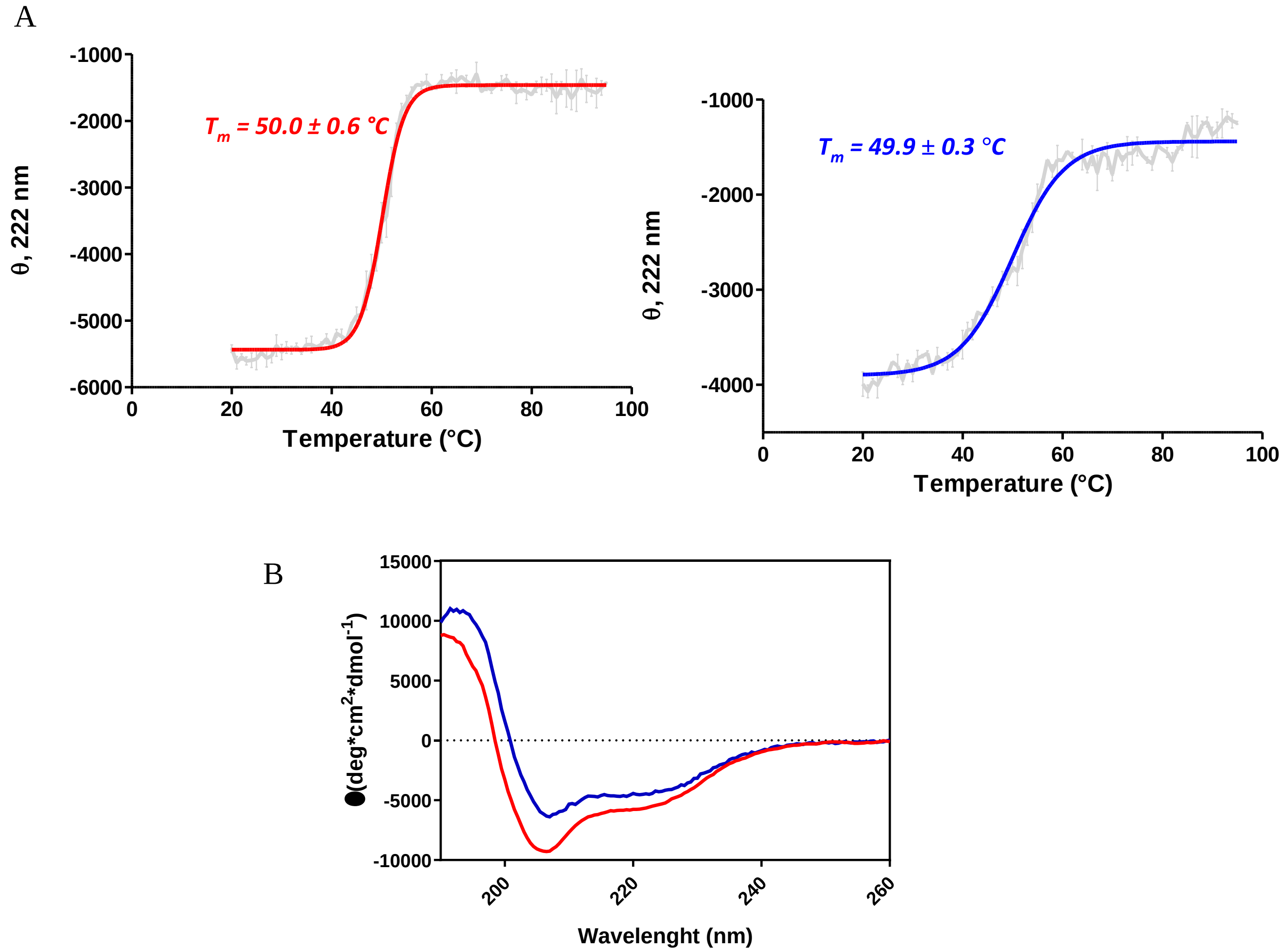

Supplementary Figure 9. (A) Thermal stability analysis: comparison of θ at 222 nm of FL-RAD52 (red) and RAD52 N-terminal (blue) for melting temperature (T_m) determination (B) Secondary structure determination of FL-RAD52 (red) and RAD52 N-terminal domain (blue) through CD spectra comparison: FL-RAD52 and N-terminal have similar absolute composition in α-helices (9% and 15%, respectively) while FL-RAD52 has more

disordered regions (51% and 32%, respectively), a reduction in β-sheets composition (7% and 30%, respectively) and an increase in turns composition, with respect to RAD52 N-terminal domain (31% and 22%, respectively).

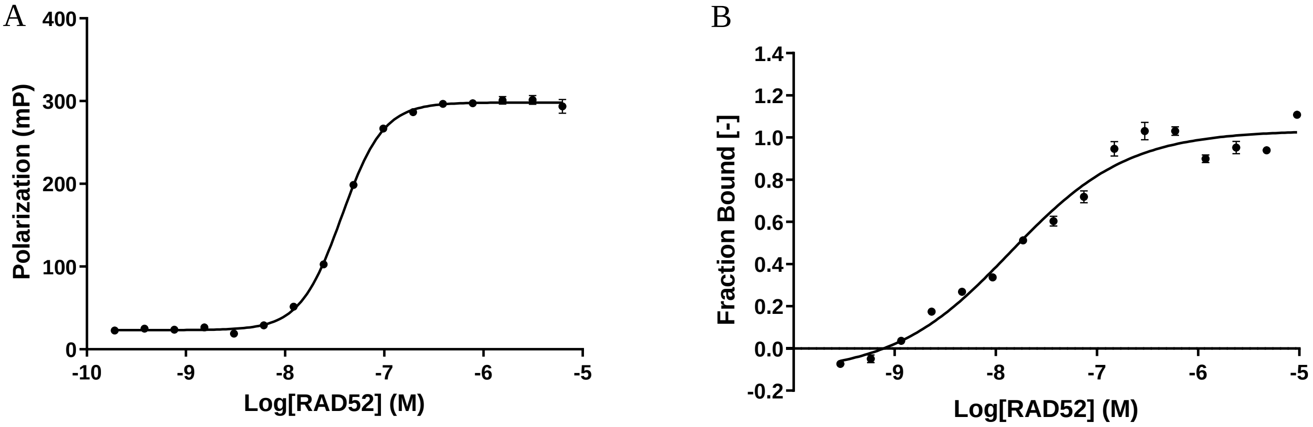

Supplementary Figure 10. FL-RAD52 DNA binding and self-oligomerization. (A) Direct FP assay of ssDNA-RAD52 binding. The plot reports polarization data (mP) recorded upon addition of increasing FL-RAD52 concentrations (Log[RAD52]) into a solution containing 10 nM 5'-FAM labeled ssDNA. The fitting curve was determined using non-linear regression (apparent K_d = 37.2 ± 0.4 nM). (B) MST analysis of protein-protein titration showing FL-RAD52 tendency to self-oligomerize. The fitting curve was determined using non-linear regression (apparent K_d = 14.2 ± 4.0 nM). The data are the average of 2 replicates.

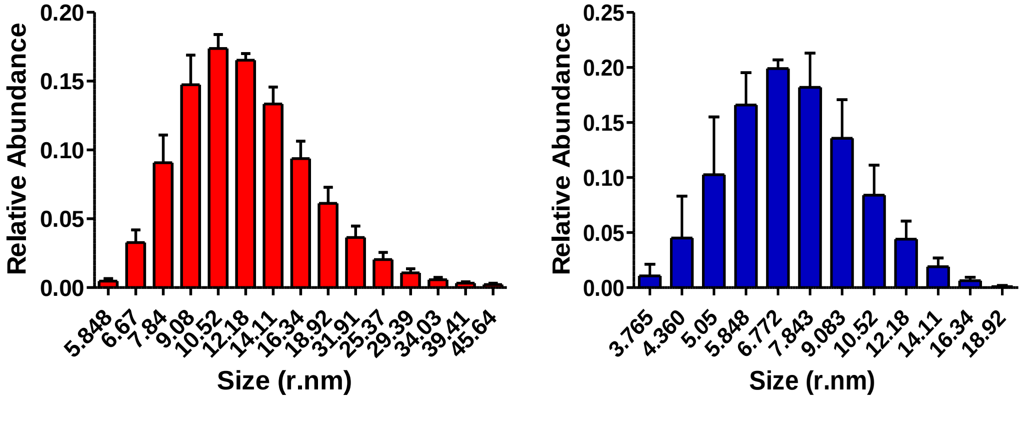

Supplementary Figure 11. DLS profile of FL-RAD52 protein size analysis (red histogram) shows a heterogeneous protein sample with high molecular weight FL-RAD52 superstructures, which are on average larger compared to the ones observed in the DLS profile of RAD52 N-terminal domain protein sample (blue histogram).

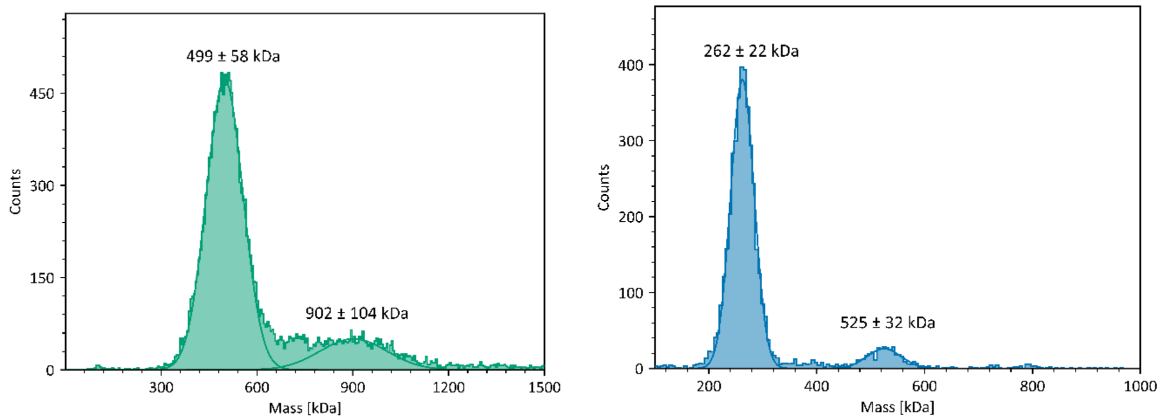

Supplementary Figure 12. Mass Photometry analysis of RAD52 FL (left) and RAD52 N-terminal domain (right) at 800 nM.

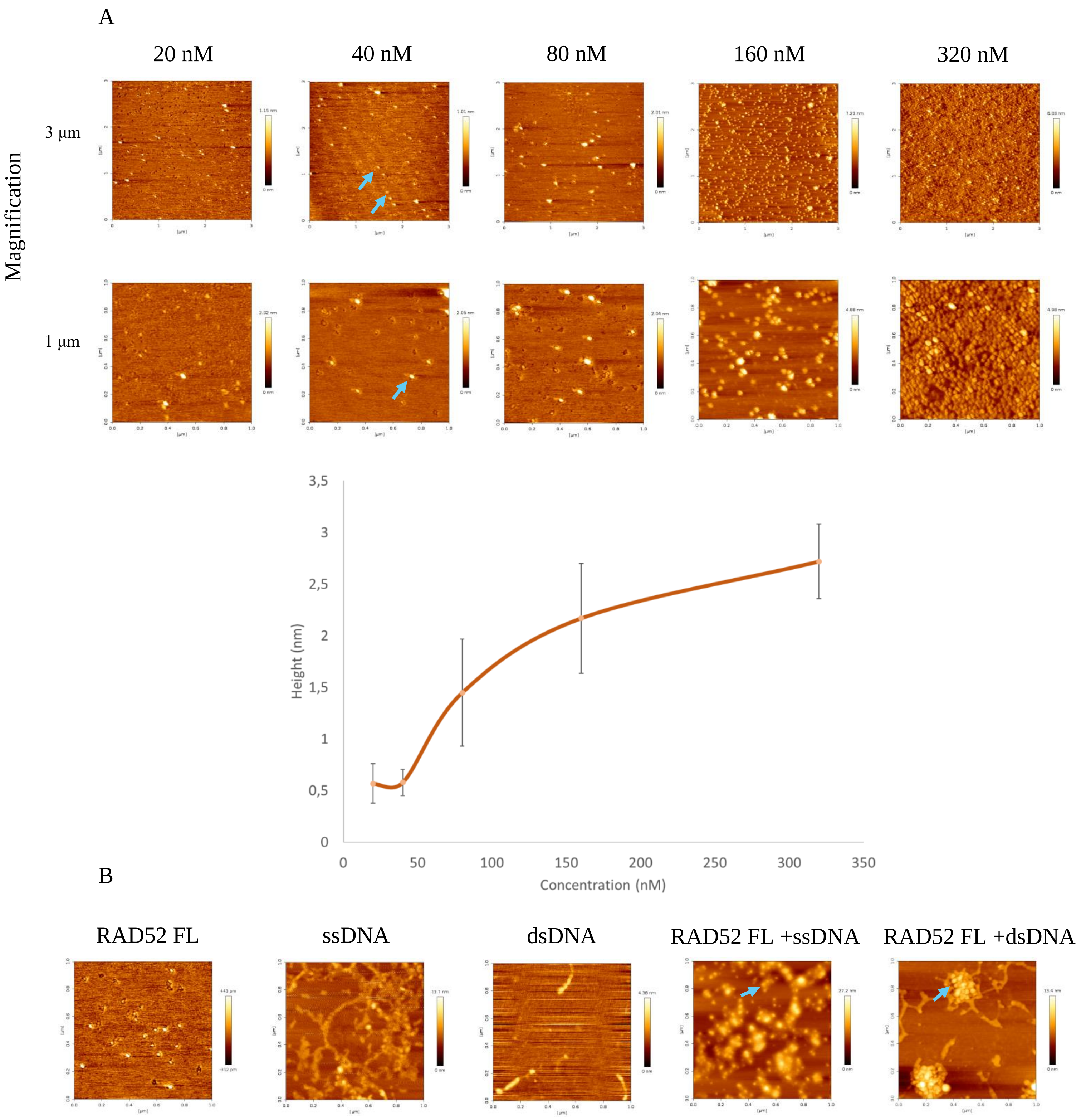

Supplementary Figure 13. (A) AFM analysis of FL-RAD52 20 nM, 40 nM, 80 nM, 160 nM, 320 nM, using two different magnifications. The graph shows the height of FL-RAD52 particles as the concentration increases. (B) AFM analysis of FL-RAD52 (40 nM) in the presence or absence of ss- and dsDNA. The resulting micrographs were compared with those of DNA and protein alone. The blue arrows highlight the FL-RAD52 particles observed in the absence and presence of ss- and dsDNA for the samples at 40 nM FL-RAD52 concentration.

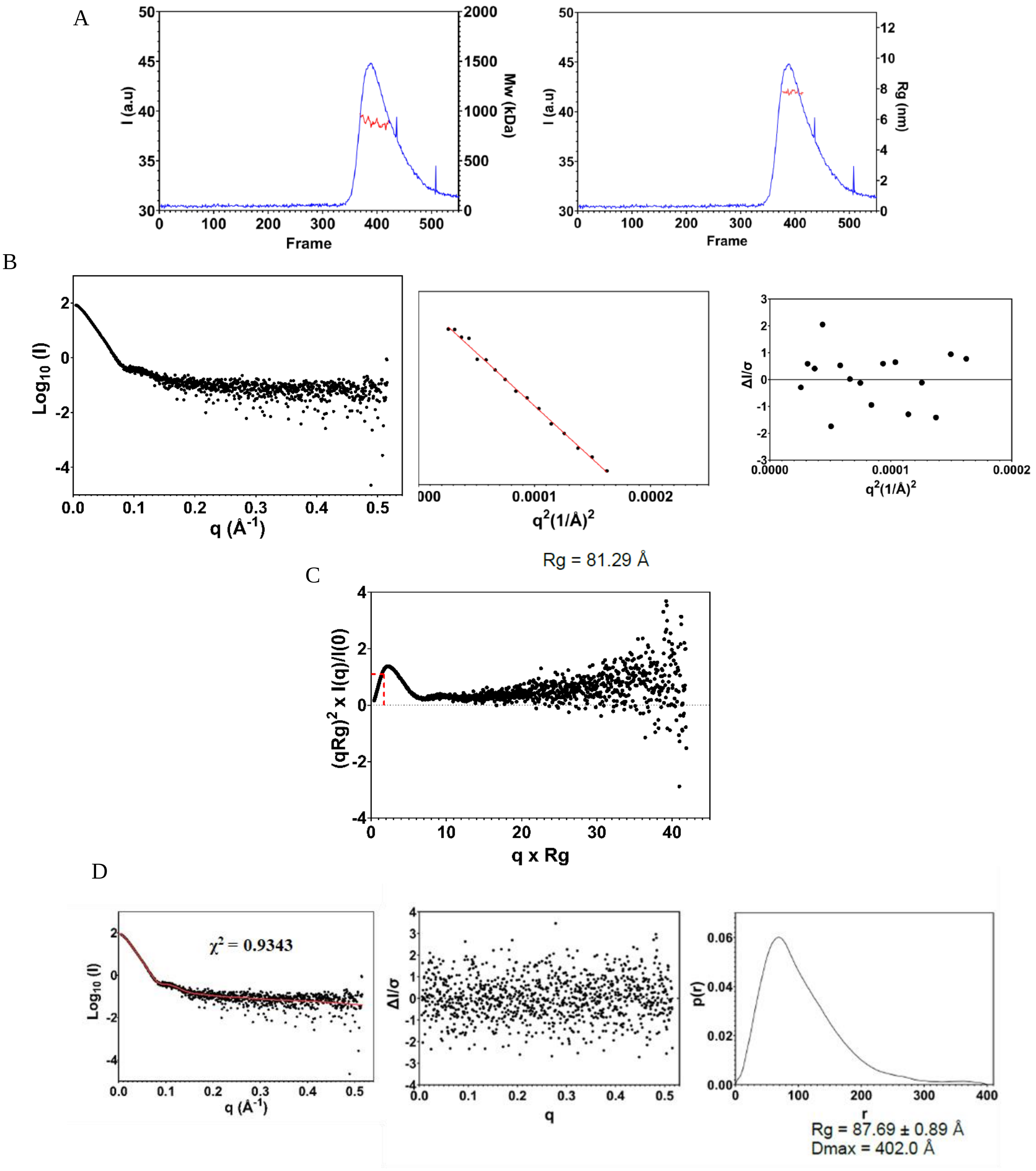

Supplementary Figure 14. (A) SEC - SAXS profile of FL-RAD52 FL, left average of mw, right average of Radius of Gyration. (B) Left: scatter profile, Center: Guiner analysis of SAXS Profile Right: Residuals of Guinier analysis. (C) Dimensionless Kratky Plot. (D) P(r) analysis.

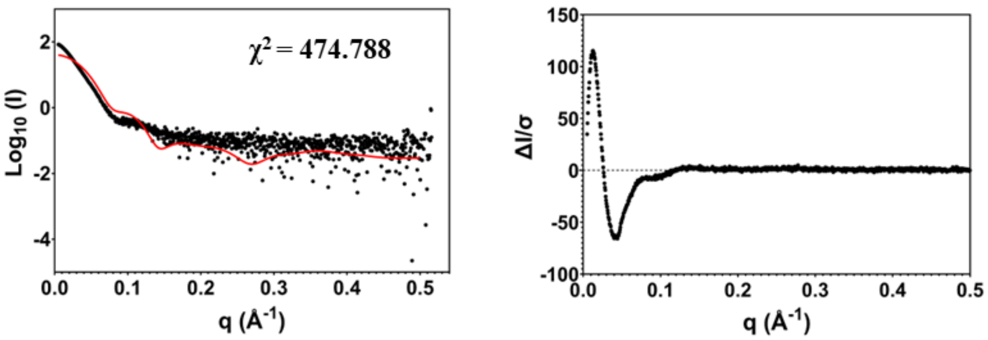

Supplementary Figure 15. FoXS modelling for the Cryo-EM structure (8BJM, red curve) compared to the experimental SAXS data obtained for the FL-RAD52.

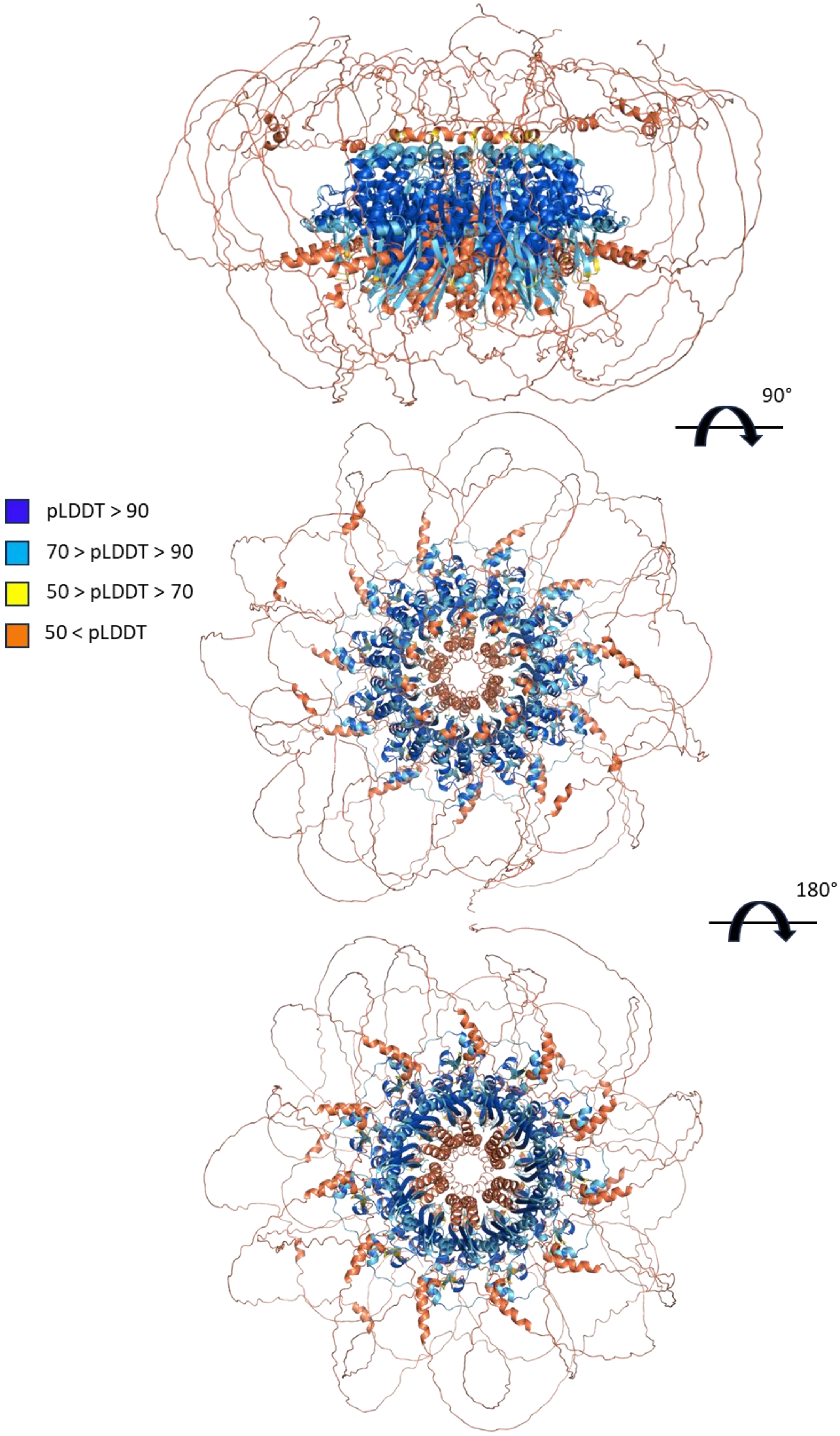

Supplementary Figure 16. Three different projections the generated Alphafold 2.3 model coloured for confidence predicted local distance difference test (pLDDT).

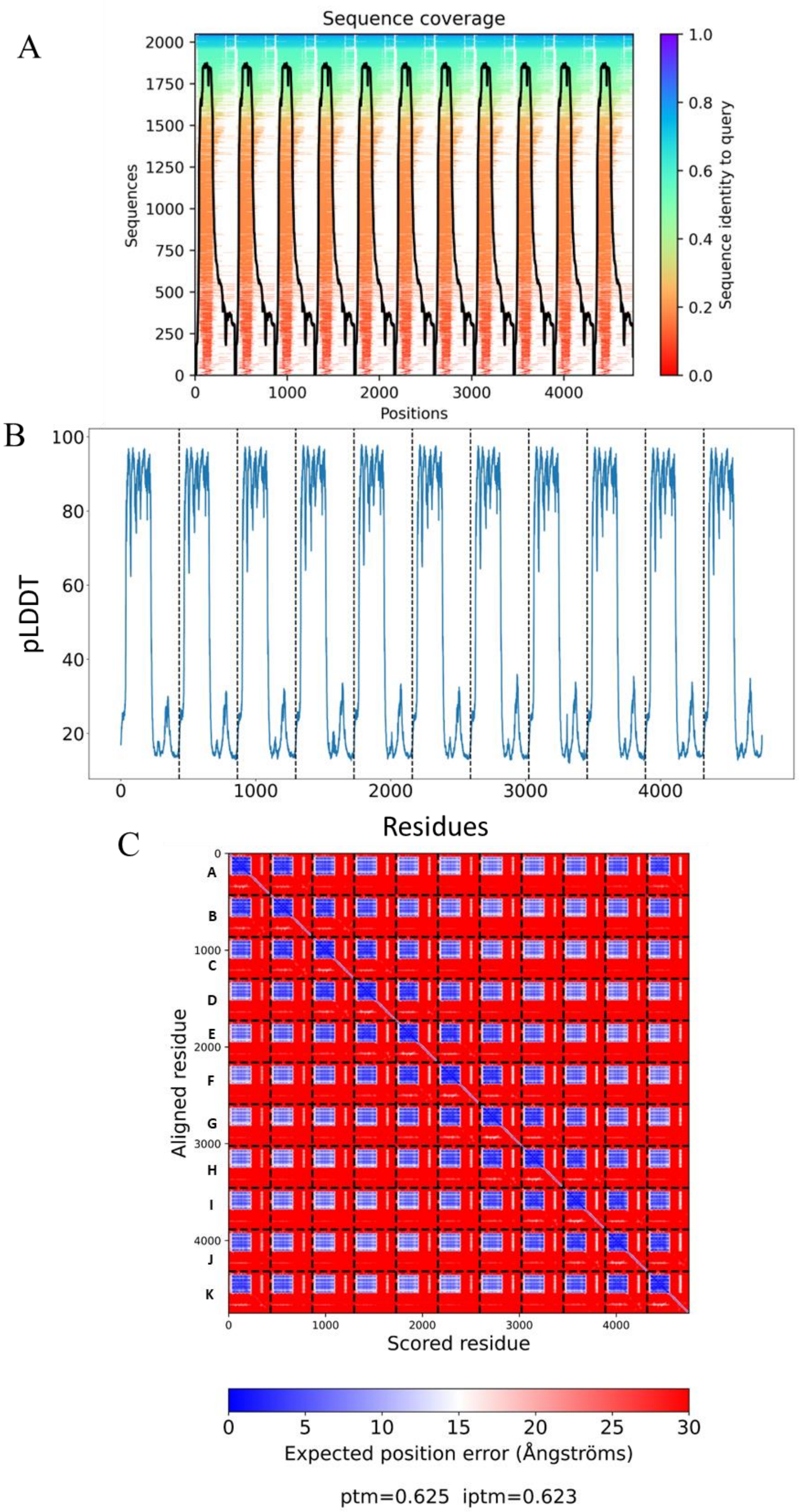

Supplementary Figure 17. (A) Multi Sequence Analysis (MSA) depth and diversity. (B) AlphaFold2 confidence predicted local distance difference test (pLDDT). (C) Predicted Align Error. Interface predicted template modelling score and predicted modelling score are reported under the plot.

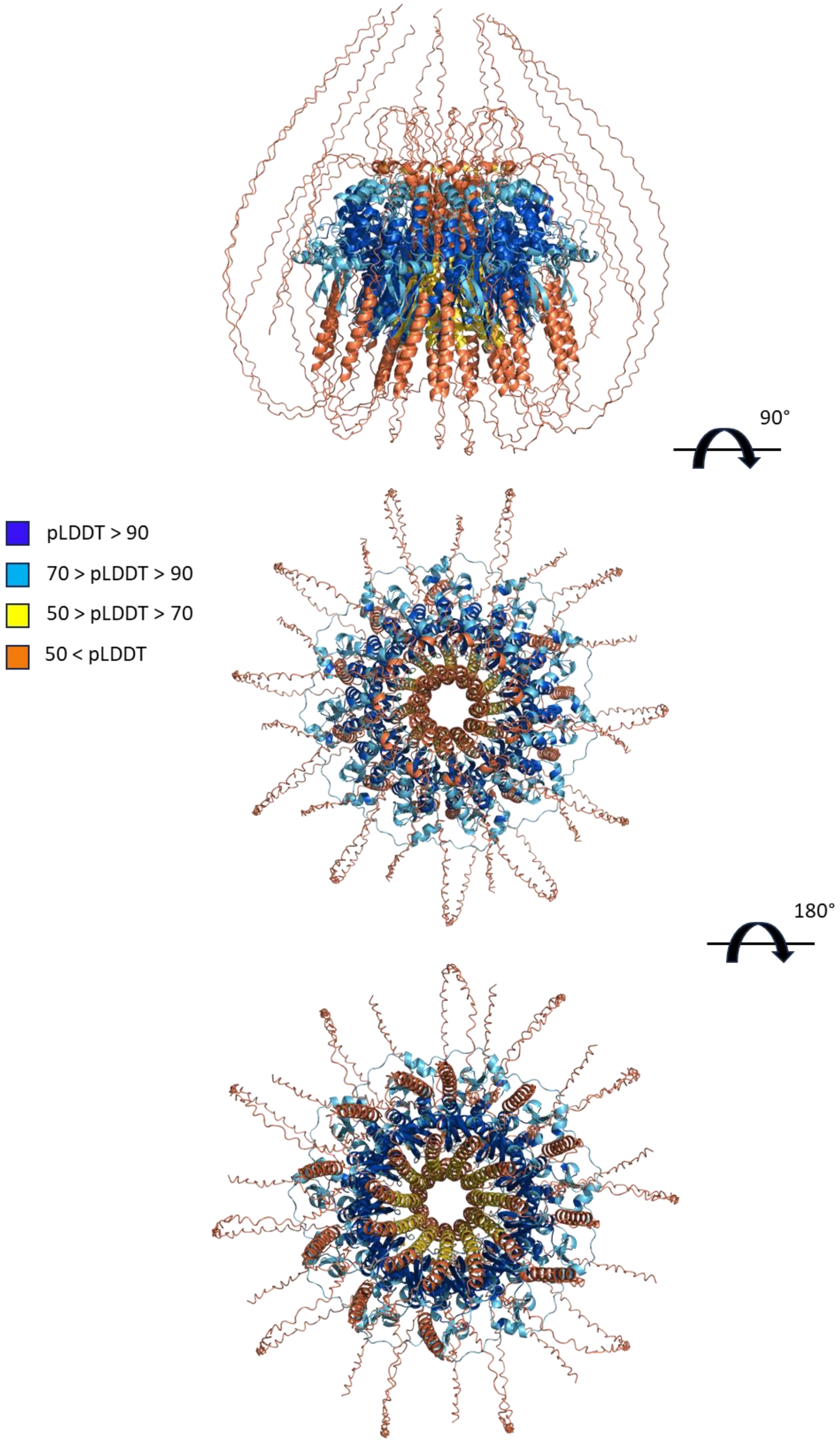

Supplementary Figure 18 Three different projections the generated Alphafold 3 model coloured for confidence predicted local distance difference test (pLDDT).

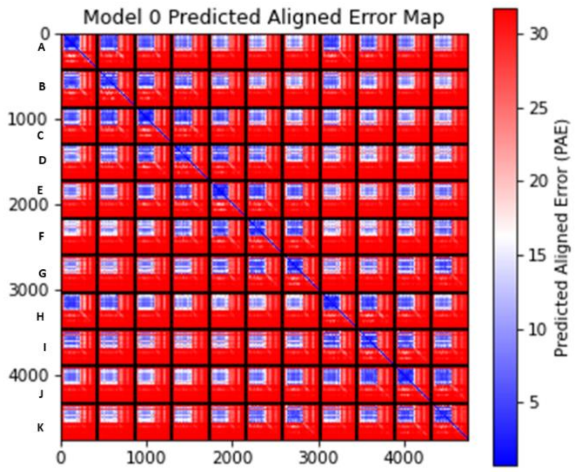

ptm = 0.58   iptm = 0.57

Supplementary Figure 19 Predicted Align Error for AF3 model. Interface predicted template modelling score and predicted modelling score are reported under the plot.

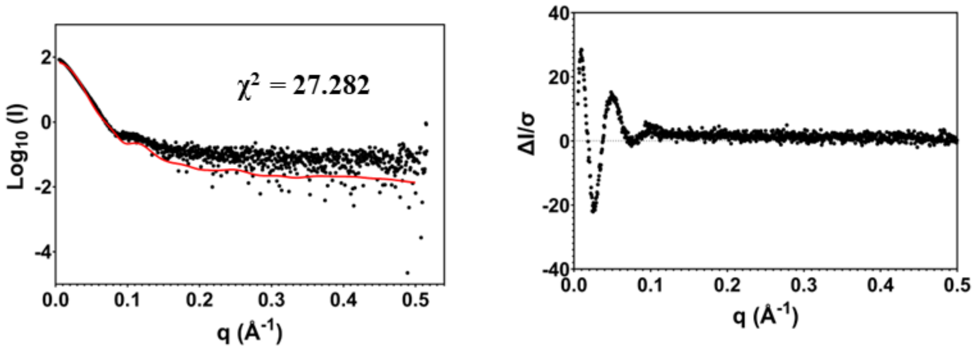

Supplementary Figure 20. FoXS modelling for the AF model (red curve) compared to the experimental SAXS data obtained for the FL-RAD52.

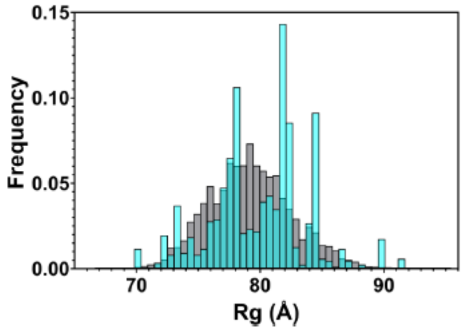

Supplementary Figure 21. EOM Modelling of the N-terminal and C-terminal domain of FL-RAD52 utilizing as a rigid body the Cryo-EM structure. Comparison of distribution of radius of gyration in selected models (cyan) and generated pool (grey).

Table S1 - Resolutions of the published cryo-EM and X-ray structures of the full-length and N-terminal RAD52.

Sample	Organism	Method	PDB accession ID	Resolution (Å)	Year of release	Reference
RAD52 N-terminal (apo)	Homo sapiens	X-ray diffraction	1KN0	2.8	2002	11
RAD52 N-terminal (apo)	Homo sapiens	X-ray diffraction	1H2I	2.7	2002	12
RAD52 N-terminal (apo)	Homo sapiens	X-ray diffraction	5JRB	2.4	2016	13
RAD52 N-terminal (bound to ssDNA)	Homo sapiens	X-ray diffraction	5XRZ	3.6	2018	14
RAD52 N-terminal (bound to ssDNA)	Homo sapiens	X-ray diffraction	5XS0	3	2018	14
RAD52 full length (apo)	Homo sapiens	Single Particle Cryo-EM	8H1P	3.5	2023	14

Table S2 - Residues involved in the FL-RAD52 intra-protomer hydrogen bonds. The residues in bold are those occurring in the large majority of protomers.

Water mediated H-bonds			Protomers										
N	Residues involved		Chain A	Chain B	Chain C	Chain D	Chain E	Chain F	Chain G	Chain H	Chain I	Chain J	Chain K
1	LEU 43 (α-helix α1)	PHE 158 O (α-helix α3)	Y	Y	Y	Y	Y	N	N	Y	N	Y	Y
2	**SER 53** (β-sheet β1)	**TYR 51** (loop L2)	Y	Y	Y	Y	Y	Y	Y	Y	Y	Y	Y
3	**ASN 76** (α-helix α2)	**TYR 81** (loop L5)	Y	Y	Y	Y	Y	Y	N	Y	Y	Y	Y
4	**SER 87** (β-sheet β3)	**ARG 112** (β-sheet β4)	Y	Y	Y	Y	Y	Y	N	Y	Y	Y	Y
5	PHE 103 (β-sheet β4)	SER 138 OG (α-helix α3)	Y	N	Y	N	Y	N	N	Y	Y	N	Y
6	ASP 123 (α-helix α3)	ARG 156 (α-helix α3)	Y	N	Y	N	Y	Y	Y	Y	N	Y	Y
7	LEU 132 (loop L8)	SER 138 (α-helix α3)	Y	N	N	N	Y	N	N	Y	Y	N	Y
8	LYS 152 (α-helix α3)	ARG 156 (α-helix α3)	N	Y	Y	N	Y	N	Y	Y	Y	Y	Y
9	ARG 156 (α-helix α3)	SER 157 (loop L9)	N	N	Y	N	Y	N	Y	N	N	N	N
10	ASN 160 (loop L9)	LEU 43 (α-helix α1)	Y	Y	Y	Y	Y	N	N	Y	N	Y	Y
11	**ASN 160** (loop L9)	**PHE 158** (loop L9)	Y	Y	Y	Y	Y	Y	N	Y	Y	Y	Y
12	LEU 162 (loop L9)	ARG 156 (α-helix α3)	N	Y	Y	N	Y	N	Y	Y	Y	Y	Y
13	**LEU 162** (loop L9)	**LYS 152** (α-helix α3)	Y	Y	Y	Y	Y	Y	Y	Y	Y	Y	Y

Table S3 - Residues involved in the FL-RAD52 inter-protomer hydrogen bonds. The residues in bold are those occurring in all protomers.

Water mediated hydrogen bond			hFLRAD52 chains										
N	Residues involved		Chains A-B	Chains B-C	Chains C-D	Chains D-E	Chains E-F	Chains F-G	Chains G-H	Chains H-I	Chains I-J	Chains J-K	Chains K-A
1	**ASN 76** (α-helix α2, protomer n)	**HIS 121** (β-sheet β5, protomer n+1)	Y	Y	Y	Y	Y	Y	Y	Y	Y	Y	Y
2	**TYR 81** (β-sheet β3, protomer n)	**HIS 121** (β-sheet β5, protomer n+1)	Y	Y	Y	Y	Y	Y	Y	Y	Y	Y	Y
3	**ASN 82** (loop L5, protomer n)	**ASP 117** (loop L7, protomer n+1)	Y	Y	Y	Y	Y	Y	Y	Y	Y	Y	Y
4	TRP 84 (β-sheet β3, protomer n)	HIS 121 (β-sheet β5, protomer n+1)	Y	N	N	N	N	N	Y	N	N	N	N
5	HYS 86 (β-sheet β3, protomer n)	ASP 123 (β-sheet β5, protomer n+1)	Y	N	Y	Y	N	N	Y	Y	Y	Y	N
6	**SER 87** (protomer n)	**TYR 120** (β-sheet β5, protomer n+1)	Y	Y	Y	Y	Y	Y	Y	Y	Y	Y	Y
7	ARG 112 (β-sheet β4, protomer n)	TYR 120 (β-sheet β5, protomer n+1)	Y	Y	Y	Y	Y	Y	Y	Y	Y	N	Y
8	**LYS 190** (loop L10, protomer n)	**ARG 46** (loop L2, protomer n+1)	Y	Y	Y	Y	Y	Y	Y	Y	Y	Y	Y
9	Glu(197) (loop L10, protomer n)	Ala(38)(α-helix α1, protomer n+1)	Y	Y	Y	N	N	Y	Y	N	Y	N	N
10	Ala(203) (α-helix α5, protomer n)	Thr(32) (loop L1, protomer n+1)	Y	N	Y	Y	N	Y	Y	Y	Y	N	Y

Table S4 - RMSD FL-RAD52 cryoEM model available crystallographic structures

RAD52 Models	RMSD
$RAD52_{25\text{-}208}$ (PDB ID: 1KN0)	0.625
$RAD52_{25\text{-}208}$ inner DNA binding site (PDB ID: 5XRZ)	0.547
$RAD52_{25\text{-}208}$ outer DNA binding site (PDB ID: 5XS0)	0.539

Table S5 - Per-Residue C-α deviations

Per-Residue C-α deviations				
PDB	8H1P	1KN0	5XZR	5XS0
RMSD (Å)	0.60	0.57	0.56	0.50
Single residue C-α Deviations Average	0.47	0.40	0.40	0.34
Highest single residue C-α deviations	3.1	4.2	4.0	1.6

Table S6 - SAXS Data Collection

(a) Sample details

Organism	Homo sapiens (Human)
Source	*E. Coli* expressed
Uniprot sequence ID	P43351
Extinction coefficient [A_{280}, 0.1%(w/v)]	0.873
$\bar{v}$ from chemical composition (cm^3/ g)	0.726 cm^3/g
Particle contrast from sequence and solvent constituents $\Delta\rho$ $10^{10}cm^{-2}$	2.985
M from chemical composition (Da)	527540
SEC–SAXS column	Superose™ 6 Increase 3.2/300
Loading concentration (mg/mL)	1.3
Injection Volume (mL)	100 µL
Flow rate (mL/min)	0.075 mL/min
Solvent (solvent blanks taken from SEC flow-through prior to elution of protein)	25 mM Tris pH 7.5, 250 mM NaCl, 1% Glycerol

(b) SAXS data-collection parameters

Instrument/data processing	FreeSAS[1]
Wavelength (Å)	0.99 (12.5KeV)
Beam size at sample (mm)	0.200 × 0.100 at sample plane
Beam size at detector (focus) (mm)	1 pixel ~ 0.1 x 0.1
Camera length (m)	2.81
q measurement range (Å)	0.007–0.55
Absolute scaling method	Water
Sample configuration	SEC-SAXS
Sample temperature (°C)	20
Exposure time	2 second / frame (600 frames)

(c) Software employed for SAXS data reduction, analysis and interpretation

SAXS data reduction	FreeSAS[1] Solvent subtraction and frame selection were performed using Chromixs (ATSAS 3.1.1) [2,3]

Extinction coefficient estimate	Expasy ProtParam tool [4]
Calculation of $\bar{v}$ and $\bar{\rho}$ values	MULCh: ModULes for the analysis of Contrast variation data (https://smb-research.smb.usyd.edu.au/NCVWeb/)
Basic analyses: Guinier, P(r), VP	BioXTAS RAW, GNOM [5,6]
Atomic structure modelling	RANCH via ATSAS Online (https://www.embl-hamburg.de/biosaxs/atsas-online/)[7,8]<br>FFMAKER, ATSAS 3.1.1 (run on local Pc)[7,8]<br>GAJOE 2.1, ATSAS 3.1.1 (run on local Pc)[7,8]
3D Models Generation	Alphafold2.3 run as a Singularity Container on IIT-Franklin Cluster, no amber relaxation enabled.

(d) Structural parameters

Guinier analysis	
I(0) (cm^{-1})	88.51 ± 0.34
R_g (Å)	81.29
q_{min} ($Å^{-1}$)	0.00507
qR_g max	1.04
Coefficient of correlation, R^2	0.9957
P(r) analysis	
I(0) (cm^{-1})	89.86 ± 0.36
R_g (Å)	87.69 ± 0.89
dmax (Å)	402.0
q range ($Å^{-1}$)	0.0051 – 0.5156
χ^2 (total estimate from GNOM)	0.9343
GNOM Interpretation	A Reasonable Solution
Porod volume ($Å^{-3}$)	1330000
V, M using the Fischer method	818000, 678.7 kDa
V_c, M	592.9 kDa
Bayesian inference	585.2 kDa

(e) Atomistic modelling.

Structure	8BJM	Complete AF 2.3 RAD52
q range modelling	0.0507 – 0.5	0.0507 – 0.5
FoXS		
Predicted R_g	40.26 Å	63.35
c_1, c_2	1.05, 4.00	1.01, -0.13
χ^2	464.79	27.28
Multistate/ensemble models		
Ensemble Optimization Method [7,8] (default parameters, 20000 models in initial ensemble, disordered models, constant subtraction, curve repetition in ensemble, maximum number of curves per ensemble 20, minimum number of curves per ensemble 5, curve repetition in the ensemble allowed, number of cycles of the genetic algorithm to run (min. 1): 100		

Structure	PDB Entry: 8BJM
q range for all modelling (Å)	0.0507 – 0.5
χ^2, CORMAP P-value	0.960, 0.208
Constant subtraction	0.058
No. of representative structures	4

(f) SASBDB IDs for data and models[9]

His-FL-RAD52	SASDQ49 – His-Tagged full length DNA repair protein FL-RAD52 homolog

(g) ESRF - DOI

All data are saved in hierarchical data format (HDF) will be available at [10]

Table S7 - Cryo EM Data Collection

	Screening session	High-resolution Session
Microscope model	Thermo Fisher Scientific Glacios Selectris X	Thermo Fisher Scientific IC-Krios Selectris X
Detector type	Thermo Fisher Scientific Falcon 4 EC	Thermo Fisher Scientific Falcon 4 EC
Imaging mode	EF-TEM	EF-TEM
Accelerating voltage, kV	200	300
Pixel size, Å	1.154 Å/pix	0.731 Å/pix
Total exposure time, sec	8.6 sec	8 e^-/pix/sec
Total Number of collected stacks	831	17400
Number of stacks used in the analysis	831	17400
Total dose per stack, e−/Å2	40.9 $e^-/Å^2$	50 $e^-/Å^2$
Number of frames per stack	34	49
Defocus range, µm	-1.2 um to -2.5 um	-0.8 um to -1.8

Table S8 - Data Processing, Global Resolution (Å), PDB ID and EMDB ID

	Screening session	High-resolution Session
3D reconstruction software package	Relion 3.1	Relion 4.0
Extracted particles	580952	2325722
Refined particles	221976	837272
Symmetry	C11	C11
FSC0.143 (unmasked/masked)	3.4 Å/pix	2.16 Å/pix
PDB ID	-	8BJM
EMBD ID	-	EMD-16089

References

1. Tully, M. D. *et al.* BioSAXS at European Synchrotron Radiation Facility – Extremely Brilliant Source: BM29 with an upgraded source, detector, robot, sample environment, data collection and analysis software. *J. Synchrotron Radiat.* **30**, 1–9 (2023).

2. Panjkovich, A. & Svergun, D. I. CHROMIXS: automatic and interactive analysis of chromatography-coupled small-angle X-ray scattering data. *Bioinformatics* **34**, 1944–1946 (2018).

3. Manalastas-Cantos, K. *et al.* ATSAS 3.0 : expanded functionality and new tools for small-angle scattering data analysis. *J. Appl. Crystallogr.* **54**, 343–355 (2021).

4. Gasteiger, E. *et al.* The Proteomics Protocols Handbook. *Proteomics Protoc. Handb.* 571–608 (2005) doi:10.1385/1592598900.

5. Hopkins, J. B., Gillilan, R. E. & Skou, S. BioXTAS RAW: Improvements to a free open-source program for small-angle X-ray scattering data reduction and analysis. *J. Appl. Crystallogr.* **50**, 1545–1553 (2017).

6. Svergun, D. I. Determination of the regularization parameter in indirect-transform methods using perceptual criteria. *J. Appl. Crystallogr.* **25**, 495–503 (1992).

7. Tria, G., Mertens, H. D. T., Kachala, M. & Svergun, D. I. Advanced ensemble modelling of flexible macromolecules using X-ray solution scattering. *IUCrJ* **2**, 207–217 (2015).

8. Bernadó, P., Mylonas, E., Petoukhov, M. V., Blackledge, M. & Svergun, D. I. Structural characterization of flexible proteins using small-angle X-ray scattering. *J. Am. Chem. Soc.* **129**, 5656–5664 (2007).

9. Kikhney, A. G., Borges, C. R., Molodenskiy, D. S., Jeffries, C. M. & Svergun, D. I. SASBDB: Towards an automatically curated and validated repository for biological scattering data. *Protein Sci.* **29**, 66–75 (2020).

10. Rinaldi, F., Hočevar, J. & Scietti, L. Proteins related to pathogenesis of diseases, viral proteins, cell division, signalling and chromatin processes [Dataset]. *European Synchrotron Radiation Facility* (2025) doi:https://doi.org/10.15151/ESRF-ES-771426690.

11. Kagawa, W., Kurumizaka, H., Ikawa, S., Yokoyama, S. & Shibata, T. Homologous Pairing Promoted by the Human, Rad52 Protein. Journal of Biological Chemistry 276, 35201–35208 (2001).
12. Singleton, M. R., Wentzell, L. M., Liu, Y., West, S. C. & Wigley, D. B. Structure of the single-strand annealing domain of human RAD52 protein. *Proc Natl Acad Sci U S A* **99**, 13492–13497 (2002).
13. Saotome, M., Saito, K., Onodera, K., Kurumizaka, H., Kagawa, W. Structure of the human DNA-repair protein RAD52 containing surface mutations. (2016) Acta Crystallogr F Struct Biol Commun 72: 598-603
14. Saotome, M. *et al.* Structural Basis of Homology-Directed DNA Repair Mediated by RAD52. *iScience* **3**, 50–62 (2018).
