# Supplementary material for: An integrative structural study of the human full-length RAD52 at 2.2 Å resolution
Source: Commun Biol. 2024 Aug 8;7:956. doi: 10.1038/s42003-024-06644-1 (PMC11306251; doi:10.1038/s42003-024-06644-1)
Supplement: Supplementary file 2 — Reporting Summary [file 42003_2024_6644_MOESM2_ESM.pdf]

nature portfolio

Corresponding author(s): Andrea Cavalli, Stefania Girotto

Last updated by author(s): 05/07/2024

Reporting Summary

Nature Portfolio wishes to improve the reproducibility of the work that we publish. This form provides structure for consistency and transparency in reporting. For further information on Nature Portfolio policies, see our Editorial Policies and the Editorial Policy Checklist.

Statistics

For all statistical analyses, confirm that the following items are present in the figure legend, table legend, main text, or Methods section.

n/a	Confirmed	
☐	☒	The exact sample size (*n*) for each experimental group/condition, given as a discrete number and unit of measurement
☐	☒	A statement on whether measurements were taken from distinct samples or whether the same sample was measured repeatedly
☒	☐	The statistical test(s) used AND whether they are one- or two-sided<br>*Only common tests should be described solely by name; describe more complex techniques in the Methods section.*
☒	☐	A description of all covariates tested
☒	☐	A description of any assumptions or corrections, such as tests of normality and adjustment for multiple comparisons
☐	☒	A full description of the statistical parameters including central tendency (e.g. means) or other basic estimates (e.g. regression coefficient) AND variation (e.g. standard deviation) or associated estimates of uncertainty (e.g. confidence intervals)
☒	☐	For null hypothesis testing, the test statistic (e.g. *F*, *t*, *r*) with confidence intervals, effect sizes, degrees of freedom and *P* value noted<br>*Give P values as exact values whenever suitable.*
☐	☒	For Bayesian analysis, information on the choice of priors and Markov chain Monte Carlo settings
☐	☒	For hierarchical and complex designs, identification of the appropriate level for tests and full reporting of outcomes
☒	☐	Estimates of effect sizes (e.g. Cohen's *d*, Pearson's *r*), indicating how they were calculated

Our web collection on statistics for biologists contains articles on many of the points above.

Software and code

Policy information about availability of computer code

Data collection	SerialEM 4.1, FreeSAS, Mo.Control, AcquireMP
Data analysis	Relion3.1, Relion4.0, MotionCor2, CTFFIND 4.1, Phenix suite (Phenix real-space refinement, phenix.douse), COOT, UCSF Chimera, UCSF ChimeraX, 2StrucCompare, DiscoverMP, Pymol, Atsas 3.1.1, PRIMUS, GNOM, AutoRg, Chromis, FFMAKER, GAJOE, BioXTAS RAW, ATSAS online, Alphafold 2.3, Alphafold 3, MO. Affinity Analysis, Zetasizer Nanoparticles Analyzer Softwar (Malvern), Graphpad PRISM, Clustal Omega, Jalview

For manuscripts utilizing custom algorithms or software that are central to the research but not yet described in published literature, software must be made available to editors and reviewers. We strongly encourage code deposition in a community repository (e.g. GitHub). See the Nature Portfolio guidelines for submitting code & software for further information.

Data

Policy information about availability of data

All manuscripts must include a data availability statement. This statement should provide the following information, where applicable:

- Accession codes, unique identifiers, or web links for publicly available datasets
- A description of any restrictions on data availability
- For clinical datasets or third party data, please ensure that the statement adheres to our policy

The protein model and the cryo-electron density map of the FL-RAD52 are available at the protein data bank (https://www.rcsb.org/) with the following accession

codes: 8BJM and emd_16089. The full wwPDB EM Validation Report (D_1292126131_val-report-full_P1.pdf.V15_4msReview.pdf) is available as attached material. SAXS data and EOM models are openly available in SASBDB with reference number SASDQ49

Research involving human participants, their data, or biological material

Policy information about studies with human participants or human data. See also policy information about sex, gender (identity/presentation), and sexual orientation and race, ethnicity and racism.

Reporting on sex and gender	Not applicable.
Reporting on race, ethnicity, or other socially relevant groupings	Not applicable.
Population characteristics	Not applicable.
Recruitment	Not applicable.
Ethics oversight	Not applicable.

Note that full information on the approval of the study protocol must also be provided in the manuscript.

Field-specific reporting

Please select the one below that is the best fit for your research. If you are not sure, read the appropriate sections before making your selection.

☒ Life sciences ☐ Behavioural & social sciences ☐ Ecological, evolutionary & environmental sciences

For a reference copy of the document with all sections, see nature.com/documents/nr-reporting-summary-flat.pdf

Life sciences study design

All studies must disclose on these points even when the disclosure is negative.

Sample size	For the cryo-EM single particle analysis, data screening and high-resolution data acquisition for cryo-EM single particle analysis were performed respectively on 831 and 17400 counting mode movies. For SAXS data collection 100 uL at 1.5 mg/mL were injected into a Superose 6 3.2/300 pre-equilibrated in running buffer and acquired at BM29 ESRF. For Fluorescence Polarization (FP) experiments dT30 ssDNA (6FAM) was tested against 16 different concentrations of FL-RAD52 ranging from 0.2 nM to 6.25 µM. For MST experiments 16 different concentrations of unlabeled FL-RAD52 ranging from 0.7 nM - 25 µM were tested.
Data exclusions	For the cryo-EM single particle analysis, as usual, we excluded bad images (i.e. micrographs on non vitreous ice, micrographs on the grid carbon layer, micrographs with few particles, micrographs that was impossible to correct for the CTF etc) and particles containing ice, damaged protein or artifacts. For SAXS data, ESRF BM29 data reduction pipeline was directly applied during data collection. Standard algorithm (Chromixs, AutoRg, Shanum, GNOM) were utilized to analyze SAXS data and obtain the range of useful points for various analysis (e.g. Rg Dmax etc.) and modeling (EOM). No data have been excluded from MST and FP analysis.
Replication	MST Data are the avarage of two (2) independent experiments. For Fluorescence Polarization each measurement was the average of three (3) independent replicates. Two (2) independently measured binding curves were recorded, averaged, and then analyzed using GraphPad Prism (Sigmoidal Dose Response fit).
Randomization	Not applicable.
Blinding	Not applicable.

Reporting for specific materials, systems and methods

We require information from authors about some types of materials, experimental systems and methods used in many studies. Here, indicate whether each material, system or method listed is relevant to your study. If you are not sure if a list item applies to your research, read the appropriate section before selecting a response.

Materials & experimental systems

n/a	Involved in the study
☒	☐ Antibodies
☒	☐ Eukaryotic cell lines
☒	☐ Palaeontology and archaeology
☐	☒ Animals and other organisms
☒	☐ Clinical data
☒	☐ Dual use research of concern
☒	☐ Plants

Methods

n/a	Involved in the study
☒	☐ ChIP-seq
☒	☐ Flow cytometry
☒	☐ MRI-based neuroimaging

Animals and other research organisms

Policy information about studies involving animals; ARRIVE guidelines recommended for reporting animal research, and Sex and Gender in Research

Laboratory animals	E. Coli BL21 (DE3) Rosetta pLysS (Sigma-Aldrich)
Wild animals	Not applicable.
Reporting on sex	Not applicable.
Field-collected samples	Not applicable.
Ethics oversight	Not applicable.

Note that full information on the approval of the study protocol must also be provided in the manuscript.

Plants

Seed stocks	Not applicable.
Novel plant genotypes	Not applicable.
Authentication	Not applicable.
